# The genome and structural proteome of an ocean siphovirus: a new window into the cyanobacterial ‘mobilome’

**DOI:** 10.1111/j.1462-2920.2009.02081.x

**Published:** 2009-11

**Authors:** Matthew B Sullivan, Bryan Krastins, Jennifer L Hughes, Libusha Kelly, Michael Chase, David Sarracino, Sallie W Chisholm

**Affiliations:** 1 Department of Civil and Environmental Engineering and Department of BiologyMIT, 48-425, Cambridge, MA 02139, USA; 2 Harvard PartnersCambridge, MA 02139, USA.; 3 Ecology and Evolutionary Biology Department, University of ArizonaTucson, AZ 85721, USA.

## Abstract

*Prochlorococcus*, an abundant phototroph in the oceans, are infected by members of three families of viruses: myo-, podo- and siphoviruses. Genomes of myo- and podoviruses isolated on *Prochlorococcus* contain DNA replication machinery and virion structural genes homologous to those from coliphages T4 and T7 respectively. They also contain a suite of genes of cyanobacterial origin, most notably photosynthesis genes, which are expressed during infection and appear integral to the evolutionary trajectory of both host and phage. Here we present the first genome of a cyanobacterial siphovirus, P-SS2, which was isolated from Atlantic slope waters using a *Prochlorococcus* host (MIT9313). The P-SS2 genome is larger than, and considerably divergent from, previously sequenced siphoviruses. It appears most closely related to lambdoid siphoviruses, with which it shares 13 functional homologues. The ∼108 kb P-SS2 genome encodes 131 predicted proteins and notably lacks photosynthesis genes which have consistently been found in other marine cyanophage, but does contain 14 other cyanobacterial homologues. While only six structural proteins were identified from the genome sequence, 35 proteins were detected experimentally; these mapped onto capsid and tail structural modules in the genome. P-SS2 is potentially capable of integration into its host as inferred from bioinformatically identified genetic machinery *int*, *bet*, *exo* and a 53 bp attachment site. The host attachment site appears to be a genomic island that is tied to insertion sequence (IS) activity that could facilitate mobility of a gene involved in the nitrogen-stress response. The homologous region and a secondary IS-element hot-spot in *Synechococcus* RS9917 are further evidence of IS-mediated genome evolution coincident with a probable relic prophage integration event. This siphovirus genome provides a glimpse into the biology of a deep-photic zone phage as well as the ocean cyanobacterial prophage and IS element ‘mobilome’.

## Introduction

Phages (viruses that infect prokaryotes) represent the largest source of uncharacterized genetic diversity in the biosphere (Pedulla *et al.*, 2003). One particular group of these phages, the ocean cyanophages, has been relatively well studied because of the global abundance of their cyanobacterial hosts (Partensky *et al.*, 1999; Waterbury *et al.*, 1979; 1986), for which a number of genome sequences are available (Rocap *et al.*, 2003; Palenik *et al.*, 2003; 2006; Dufresne *et al.*, 2003; 2008; Coleman *et al.*, 2006; Kettler *et al.*, 2007). The abundance of ocean cyanophages often covaries with cyanobacterial abundance in the wild (Waterbury and Valois, 1993; Suttle and Chan, 1994; Lu *et al.*, 2001; Marston and Sallee, 2003; Sullivan *et al.*, 2003; Muhling *et al.*, 2005). Though estimating the quantitative impact of cyanophages on mortality of their cyanobacterial hosts is challenging due to the current need to compare strain-specific cyanophage titres to total cyanobacterial counts, cyanophages are thought to be responsible for a small, but significant fraction of cell mortality (Waterbury and Valois, 1993; Suttle and Chan, 1994; Fuhrman, 2000).

Three morphologies of viruses – myo-, podo- and siphoviruses – are known to infect ocean cyanobacteria. The myovirus and podovirus cyanophage families have been relatively well characterized by morphology, host range and genomics (Waterbury and Valois, 1993; Chen and Lu, 2002; Sullivan *et al.*, 2003; 2005; Mann *et al.*, 2005), and almost universally contain homologues to their host's photosynthetic machinery, including the core reaction centre genes of the photosystem (Mann *et al.*, 2003; Millard *et al.*, 2004; Lindell *et al.*, 2004, Zeidner *et al.*, 2005; Sullivan *et al.*, 2006). The core photosynthesis reaction centre gene, *psbA*, has been shown to be expressed during infection for a podovirus (Lindell *et al.*, 2005; 2007) and a myovirus (Clokie *et al.*, 2006), and is hypothesized to play a role in cyanophage fitness (Lindell *et al.*, 2007; Bragg and Chisholm, 2008; Hellweger, 2009). Not only do these genes appear to be important for the cyanophage, but sequence analysis has shown that subsections of the phage copy can be traced back to their host genome (Sullivan *et al.*, 2006). Thus, ocean cyanophages appear to influence the evolution of cyanobacterial genomes via horizontal gene transfer events, even at the level of the core reaction centres (Zeidner *et al.*, 2005; Sullivan *et al.*, 2006).

Cyanophage studies to date have focused on lytic phages, which infect the host, use its machinery to replicate and burst the cell, releasing phage progeny. In contrast, temperate phages infect their hosts and may temporarily insert their DNA into the host genome as a prophage, which is replicated with the host genome as part of the cell cycle. Expression of prophage genes often fundamentally changes the host's physiology – a process known as lysogenic conversion (Calendar, 1988). For example, pathogen-associated toxin genes are commonly encoded by prophages (Miao and Miller, 1999; Boyd *et al.*, 2001; Wagner and Waldor, 2002), which act as a mechanism for horizontally transferring such toxins between microbial species (Banks *et al.*, 2002). More than 70% of sequenced bacterial genomes contain prophages (Canchaya *et al.*, 2003; Casjens, 2003). They often represent the primary constituent of strain-to-strain variability (Simpson *et al.*, 2000; Baba *et al.*, 2002; Beres *et al.*, 2002; Smoot *et al.*, 2002), and their genes are among the most highly expressed genes in genome-wide expression studies (Smoot *et al.*, 2001; Whiteley *et al.*, 2001).

Curiously, the genomes of currently available freshwater and marine cyanobacterial genomes lack identifiable prophage (Canchaya *et al.*, 2003; Casjens, 2003; Dufresne *et al.*, 2003; 2008; Coleman *et al.*, 2006; Kettler *et al.*, 2007), in spite of two lines of indirect evidence that suggest prophages exist in cyanobacteria. First, strain-to-strain variability in marine cyanobacterial genomes is often clustered in genomic islands with signatures of phage and mobile element activity even including phage-like integrase genes (Palenik *et al.*, 2003; Coleman *et al.*, 2006; Kettler *et al.*, 2007; DuFresne *et al.*, 2008). Second, addition of inducing agents to natural seawater communities have yielded increases in culturable *Synechococcus* cyanophage thought to be induced prophage (McDaniel *et al.*, 2002; Ortmann *et al.*, 2002).

Here, we characterize the genome and proteome of an ocean siphovirus that was isolated from 83 m deep Atlantic Ocean slope waters using *Prochlorococcus* MIT9313 as a host strain. The data are analysed on their own, and in an evolutionary context using comparative genomics of *Prochlorococcus* and *Synechococcus* genomes. To this end, we uncover basic biology of the siphovirus genome, identify a possible integration site in its host, and explore the evolutionary link between insertion sequence (IS) activity and prophage integration in the *Prochlorococcus* and related *Synechococcus* host genomes.

## Results and discussion

### The architecture of the P-SS2 particle and its genome

**Fig. 1:**
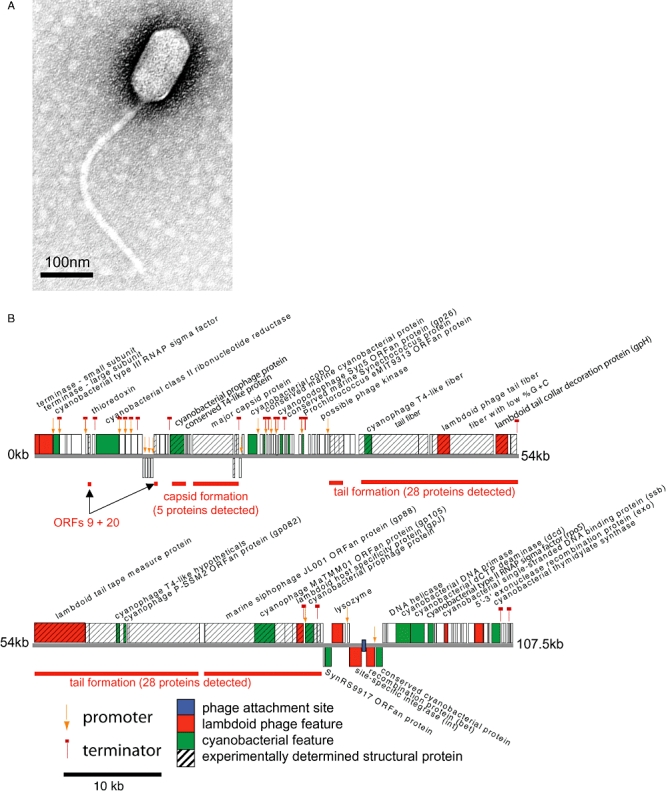
The morphology (A) and genome/structural proteome (B) of *Prochlorococcus* siphovirus P-SS2. A. Electron micrograph of uranyl acetate negative-stained, purified P-SS2 viral particle. B. The open reading frames (ORFs) are indicated on either the positive (above grey line) or negative (below grey line) DNA strand. Bioinformatically determined promoters and terminators are indicated, as is a putative host integration site (see Fig. 2). Structural proteins detected using mass-spectrometry are indicated by the diagonal lines in the corresponding ORFs, with structural modules indicated by the red lines and the text underneath the genome. For further detail, the number of virion structural peptides detected per ORF is provided in Table 2. The genome sequence is deposited in GenBank under accession #GQ334450.

P-SS2 has the morphology of a siphovirus, with a ∼75 nm diameter elongated (∼140 nm long) capsid and a ∼325 nm flexible, non-contractile tail (Fig. 1A). This is the largest siphovirus for which a complete genome has been sequenced (Table 1), and the size of its genome is also large: at 107 595 bp (Fig. 1B; Table 1) it is surpassed only by the 122 kb coliphage T5 genome (Table 1). Of the 131 predicted open reading frames (ORFs) in the P-SS2 genome, only 38 have recognizable homologues (Table 2). This is proportionally fewer than in other siphoviruses, where often half the predicted proteins have recognizable homologues (Brussow and Desiere, 2001; Proux *et al.*, 2002; Pedulla *et al.*, 2003). It is, however, proportionally similar to the alpha-proteobacterial marine siphovirus phi-JL001 where only 17 of 91 ORFs had homologous proteins in the database (Lohr *et al.*, 2005), and consistent with the idea that marine siphoviruses encode proteins that are under-represented in the database. Of the 38 ORFs in the P-SS2 genome that have homologues (Table 2), 24 have ascribed functions, eight are hypotheticals predominately from cyanobacteria or their phages, and six are ORFan proteins with a single database match.

**Table 2:** Summary table of P-SS2 predicted proteins that contained relevant annotation information as determined from (a) significant blastp hits (*e*-value < e^−3^) against the GenBank non-redundant database, (b) experimental proteomics on the virus particle, or (c) detection in viral metagenomes.

P-SS2 ORF #	Strand	LeftEnd	RightEnd	Size (aa)	Gene	Putative function	*e*-value	Average peptides detected
001	+	1	528	176	terS	Terminase – small subunit	e^−8^	0
002	+	525	2018	498	terL	Terminase – large subunit	e^−63^	0.5
003	+	2091	2732	214	Type III rpoS	Cyanobacterial type III RNAP sigma factor	e^−13^	0
005	+	3417	3608	64		Unknown protein in metagenomes	No hits	0
009	+	6004	6192	63		Structural protein	No hits	1.5
010	+	6423	6713	97	Thioredoxin	Thioredoxin	e^−3^	0
011	+	6853	9423	857	nrd	Cyanobacterial class II ribonucleotide reductase	e = 0	0
014	+	10792	11481	230		Hypothetical protein	e^−5^	0
020	+	13316	13579	88		Unknown structural protein	No hits	2.5
025	+	15118	16674	519		Structural prophage protein	e^−46^	33
028	+	17092	17280	63		Conserved T4-like protein in metagenomes	e^−5^	0
030	+	17648	22147	1500		Major capsid protein	e^−18^	96.5
031	–	22144	22347	68		Unknown structural protein, also in metagenomes	No hits	1.5
032	+	22350	22535	62		Unknown structural protein	No hits	1
033	+	22567	22767	67		Unknown structural protein	No hits	2.5
036	+	23802	24836	345	cobO	Cyanobacterial *cobO*	e^−98^	0
038	+	25557	25730	58		Conserved marine cyanobacterial protein	e^−12^	0
045	+	27442	27702	87	Syn5_026	Cyanopodophage Syn5 ORFan protein (gp26)	e^−13^	0
049	+	28259	28486	76		Conserved marine *Synechococcus* protein	e^−4^	0
053	+	29861	30058	66	9313_1008	*Prochlorococcus* eMIT9313 ORFan protein	e^−3^	0
058	+	31797	32363	189	Kinase	Possible phage kinase	e^−5^	0.5
061	+	32988	33524	179		Unknown structural protein	No hits	16
062	+	33526	33993	156		Unknown structural protein	No hits	3
063	+	33993	34499	169		Unknown structural protein	No hits	5
066	+	36202	36831	210		Unknown structural protein	No hits	2.5
067	+	36831	37625	265	Fibre	Cyanophage T4-like fibre	e^−7^	9.5
068	+	37635	42854	1740	Fibre	Unknown structural protein, tail fibre	e = 0.015	6
069	+	42886	43926	347		Unknown structural protein	No hits	13
071	+	44422	44988	189		Unknown structural protein	No hits	1
072	+	44988	46352	455	Fibre	Lambdoid phage tail fibre	e^−11^	3
073	+	46354	51234	1627	Fibre	Tail fibre with low %G+C	e^−35^	5
074	+	51512	52852	447	Capsid decoration protein	Lambdoid tail collar/fibre decoration protein (gpH)	e^−45^	2
076	+	53164	53838	225		Unknown structural protein	No hits	9.5
077	+	54104	59761	1886	Tail tape measure	Lambdoid tail tape measure protein	e^−36^	102
078	+	59795	60211	139		Unknown structural protein	No hits	3
079	+	60216	63218	1001		Unknown structural protein	No hits	43
080	+	63255	63593	113		Cyanophage T4-like hypotheticals	e^−5^	9
081	+	63603	64040	146		Unknown structural protein	No hits	7.5
082	+	64040	64306	89	M2_082	Cyanophage P-SSM2 ORFan protein (gp082)	e^−6^	3.5
083	+	64460	64657	66		Unknown structural protein	No hits	2
084	+	64656	72495	2613		Unknown structural protein, also in metagenomes	No hits	43
085	+	72530	73087	186		Unknown protein in metagenomes	No hits	0
086	+	73129	78666	1846		Structural protein similar to marine siphophage JL001 ORFan protein (gp88)	e^−4^	45
087	+	78666	80936	757		Structural protein similar to cyanophage MaTMM01 ORFan protein (gp105)	e^−10^	11
088	+	80960	82057	366		Unknown structural protein	No hits	17.5
089	+	82057	82920	288		Unknown structural protein	No hits	6
090	+	82920	83348	143		Unknown structural protein	No hits	4
091	+	83401	84141	247	Host specificity	Lambdoid host specificity protein (gpJ)	e^−4^	14
092	+	84331	85251	307		Structural cyanobacterial prophage protein	e^−65^	43.5
093	+	85317	85643	109		Unknown structural protein	No hits	12.5
095	+	86065	86304	80		Unknown protein in metagenomes	No hits	0
097	–	86560	87231	224	hyp_Syn	*Syn*RS9917 ORFan protein	e^−23^	0
098	+	87302	88528	409	Lysozyme	Lysozyme	e^−11^	0
101	–	89241	90614	458	int	Site-specific integrase (int)	e^−12^	0
102	–	91145	92104	320	bet	Recombination protein (bet)	e^−15^	0
103	–	92216	92956	247		Conserved cyanobacterial protein	e^−5^	0
108	+	94460	96085	542	Helicase	DNA helicase	e^−8^	0
109	+	96089	97663	525	Primase	Cyanobacterial DNA primase	e^−58^	0.5
111	+	98065	98655	197	dcd	Cyanobacterial dCTP deaminase (dcd)	e^−19^	0
113	+	98987	99841	285	Type II rpoS	Type II RNAP sigma factor (rpoS)	e^−17^	0
114	+	99889	100242	118	ssb	Cyanobacterial single-stranded DNA binding protein (ssb)	e^−22^	0
123	+	103253	104206	318	exo	5′-3′ exonuclease recombination protein (exo)	e^−11^	0
126	+	105166	106119	318	thy1	Cyanobacterial thymidylate synthase	e^−57^	0

For each protein, the genome locus information is paired with our annotations, as well as the top *e*-value and the average number of peptides detected from three biological replicate proteomic analyses (see text and methods).

**Table 1:** Genome-wide characteristics of marine siphoviruses P-SS2 (this study) and phi-JL001 (Lohr *et al.*, 2005) relative to other recognized phage groups within the Siphoviridae. Siphoviruses are all non-enveloped and contain double-stranded DNA genomes, non-contractile, flexible tails, and are distinguished by different combinations of alleles of structural and DNA replication proteins

	Genome features			Particle features	
Phage genus^a^	Size (kb)^b^	# ORFs	%G+C	Capsid diameter (nm)	Tail (nm) – L × W
Marine, non-classified siphoviruses					
cyanophage P-SS2	108	131	52.3	75	325 × 12
Alpha-protebacteria φJL001	63	91	62	75	125 × N.D.
Lambda-likes^b^					
Enterobacteria phage λ	48.5	92	49	60	150 × 8
Enterobacteria phage HK022	40.8	57	49	51	106 × N.D.
Enverobacteria phage HK97	39.7	62	49	54	179 × N.D.
T1-likes					
Enterobacteria phage T1	48.8	78	45	60	150 × 8
Enterobacteria phage TLS	49.9	87	42	50	N.D.
Enterobacteria phage RTP	46.2	75	44	60	160 × N.D.
L5-likes					
Mycobacterium phage L5	52.3	88	62	60	135 × 8
Mycobacterium phage D29	49.1	84	63	N.D.	N.D.
Mycobacterium phage Bxb1	50.6	86	63	60	135 × N.D.
φC31-likes					
Streptomyces phage φC31	41.5	54	63	53	100 × 5
Streptomyces phage φBT1	41.8	56	62	N.D.	N.D.
N15-likes					
Enterobacteria phage N15	46.4	60	51	60	140 × 8
T5-likes					
Enterobacteria phage T5	121.7	195	39	80	180 × 9
c2-likes					
Lactococcus phage bIL67	22.2	37	35	41	98 × 9
Lactococcus phage c2	22.2	41	36	N.D.	N.D.
ψM1-likes					
Methanobacterium phage *ψM1*	26.1	31	46	55	210 × 10

a Genus as recognized by the International Committee on the Taxonomy of Viruses (van Regenmortel *et al.*, 2000) and recently described Sfi21-like siphovirus families (Proux *et al.*, 2002). There are 19 sequenced genomes currently recognized as part of the lambda supergroup. Here we present a representative genome from each major group.

b Genome sizes are from the classified siphovirus genomes from the NCBI TaxBrowser database.

**Fig. 2:**
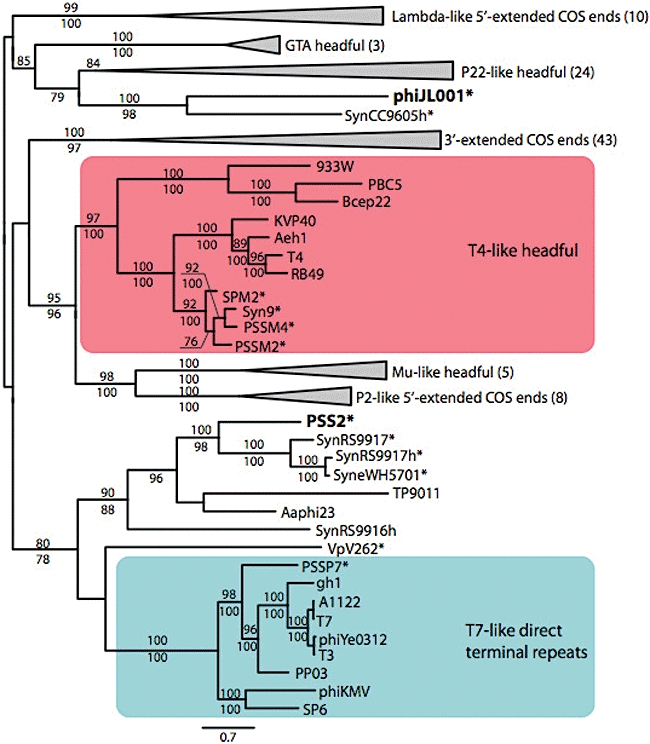
Phylogenetic relationships of the large terminase protein across diverse phage types. This protein is diagnostic of phage DNA packaging mechanisms (Casjens *et al.*, 2005) and was here used to initially characterize the P-SS2 large terminase protein relative to known phage terminases. The asterisk (*) denotes marine phage and cyanobacterial host genomes. Notably, the terminase from the other marine siphovirus whose genome is sequenced (phi-JL001) clusters separately from known terminases, while that from cyanophage P-SS2 clusters with terminases from marine cyanobacterial host genomes (likely remnant prophages, see text). The tree shown is a maximum likelihood tree constructed from 1513 positions (significantly divergent protein and gapped alignment) as described in *Experimental procedures*. Numbers above and below branches represent bootstrap values over 75 from maximum likelihood and distance analyses respectively. Numbers in parentheses with taxa labels represent number of taxa in collapsed nodes.

Twenty-two of the P-SS2 ORFs appear phage-related, with 13 of these most similar to proteins of the lambdoid siphoviruses and nine most similar to other viral types (Fig. 1B). Six have sequence homology to lambdoid structural proteins (tail fibre, tail collar *gpH*, tail tape measure, host specificity *gpJ*), recombination (*bet*), and lysis (lysozyme) proteins. Another six include ‘cyanobacterial’ analogues of lambdoid proteins (dCTP deaminase, single-stranded DNA binding protein, integrase, thymidylate synthase, and the small and large subunits of terminase) and the last encodes a ‘non-cyanobacterial’ (exonuclease, *exo*) lambdoid analogue. The remaining nine ORFs with phage-related homologues are most similar to unclassified prophage proteins (ORFs 007, 014, 025, 030, 092), T4-like myovirus proteins (ORFs 028, 067, 080), and a T7-like podovirus protein (ORF 045).

Gene expression in cyanobacteria is commonly regulated at the level of transcription, so we investigated the potential transcriptional regulatory machinery available to siphovirus P-SS2 (Fig. 1B). A search for classic sigma-70 promoter sequences and rho-independent terminators revealed 19 and 22 respectively (details in *Experimental procedures*, genome locations in Table S1 and Fig. 1B, weblogo promoter consensus sequence in Fig. S1). Notable among these were transcriptionally autonomous genes (termed ‘morons’ by Hendrix *et al.*, 1999) that are thought to be the basis for mosaicism among siphoviruses; examples here include the *Prochlorococcus* MIT9313 ORFan (P-SS2 ORF 053) and Syn5 cyanopodophage ORFan (P-SS2 ORF 045). As well, reverse promoters that were identified often provided support that predicted opposite strand ORFs may indeed be functional (e.g. P-SS2 ORFs 016–020). Sequence analysis also identified two sigma factors that are likely used by P-SS2 to modulate host RNAP activity during infection. The first, a group 2 sigma factor (ORF 113), most probably recognizes the canonical sigma-70 promoter sequences identified above, while the second, a group 3 sigma factor (ORF 003), likely recognizes sequences specific for a particular regulon (Lonetto *et al.*, 1992). Such functionally specific group 3 sigma factors are uncommon among the sequenced marine *Prochlorococcus* genomes to date; found only in *Pro*MIT9303 and the original P-SS2 host strain, *Pro*MIT9313. However, the P-SS2 group 3 sigma factor appears significantly diverged from that of its host (Fig. S2), so if the phage or host version was acquired from the other entity then it has greatly diverged or one of the two acquired the sigma factor from outside cyanobacteria and their phages.

While the bulk of P-SS2's phage-related proteins are most similar to those from lambdoid phages, P-SS2 is a distantly related lambdoid phage at best. First, the sequence similarity of the aforementioned six ‘lambdoid’ proteins is quite poor. Second, a phylogeny of the large terminase protein (diagnostic for DNA packaging characteristics; Casjens *et al.*, 2005), suggests that the P-SS2 TerL and the homologues from remnants of marine *Synechococcus* prophage integration events (see *Discussion* below) comprise a novel terminase class quite divergent from known phage terminases (Fig. 2). Third, most of the protein components that comprise the P-SS2 virus particle are unrecognizable; only six structural proteins could be assigned by sequence, as elaborated upon below.

### Structural proteins

To expand our understanding of the genes encoding the P-SS2 structure, we identified the structural proteins in purified virus particles experimentally using mass spectrometry (see *Experimental procedures*). We detected 35 structural proteins (Table 2, hashed lines in ORFs in Fig. 1B), including all six that were identified by sequence. As is common in phage genomes (Brussow and Desiere, 2001; Proux *et al.*, 2002; Casjens, 2003), these structural genes were clustered on the genome into ‘modules’ (red lines below genome in Fig. 1B). The largest cluster, consisting of 28 structural genes, included homologues to tail fibre structural genes (ORFs 067, 072, 074, 077, 091). Notably, one putative tail fibre gene (ORF 073) is predicted to encode a 1627-amino-acid protein and has a significantly low %G+C content (Fig. S3). This anomalous %G+C, and the fact that the ORF is most similar to a non-siphovirus tail fibre gene from the myovirus P-SSM2 genome, suggests possible horizontal transfer into P-SS2 from another phage class. If true, such tail fibre switching might have significant implications for host-range among cyanophages.

Another five proteins detected in the virus particle were mapped to a genome cluster that contained a capsid protein homologue (ORF 030) and a highly conserved marine prophage protein that was among the most abundant proteins in the proteomics analysis (ORF 025; averaged 33 detected peptides across biological replicates). This region likely defines proteins involved in capsid formation. The last two structural proteins detected in the virus particle are small proteins in the 5′-end of the P-SS2 genome (ORFs 009, 020) with unknown function.

### Cyanobacterial and marine features of the P-SS2 genome

The P-SS2 genome is 108 kb whereas, with one exception, most of the other siphoviruses sequenced to date have genomes on the order of 20–50 kb (Table 1). What comprises this extra DNA? Unlike the majority of cultured marine myovirus and podovirus (Mann *et al.*, 2003; Millard *et al.*, 2004; Lindell *et al.*, 2004; 2005; 2007; Sullivan *et al.*, 2006), P-SS2 does not encode cyanobacterial photosynthesis genes. Because cyanomyoviruses were commonly isolated from similar deep-photic zone depths that also contain *psbA* (12 are documented in Sullivan *et al.*, 2006), we posit that the lack of such photosynthesis genes in siphovirus P-SS2 is more likely to be due to the hypothesized temperate phage lifestyle of this virus.

However, P-SS2 does encode 14 genes with homology to genes from ocean cyanobacteria. Six of these proteins are also phage-encoded in the lambda/*Escherichia coli* system, and likely, as described above for cyanobacterial lambdoid analogues in the genome section have important DNA synthesis and packaging functions. The remaining eight, which have host but not phage parallels in the lambda/*E. coli* system, include a cyanobacterial DNA primase (paired with a phage-encoded non-cyanobacterial DNA helicase), ribonucleotide reductase (RNR), cobalamin synthesis gene *cobO*, three conserved marine cyanobacterial hypothetical proteins, and two cyanobacterial ORFan genes. The last six of these genes have not been seen previously in any phage genome, and their functional roles and importance to phage fitness remain unclear. In contrast, primase, helicase and RNR-encoding genes are common in phage genomes. While not found in lambda, primase and helicase genes are often present in other siphovirus genomes, including the divergent ocean siphovirus phi-JL001, suggesting that these genes encode critical protein functions not required in lambda. Further, while RNR-encoding genes are uncommon among siphoviruses (found in 12 of 107 siphoviruses at http://rnrdb.molbio.su.se), two lines of evidence suggest their importance in marine ecosystems. First, the other marine siphovirus, phi-JL001, contains a RNR-encoding gene (Lohr *et al.*, 2005). Second, they are also found in non-siphovirus marine phage: all marine T4-like and T7-like phage sequenced to date contain them (Rohwer *et al.*, 2000; Chen and Lu, 2002; Mann *et al.*, 2005; Sullivan *et al.*, 2005; Pope *et al.*, 2007; Weigele *et al.*, 2007) even though they are absent in non-marine T7-like phages. We hypothesize that the prevalence of RNR-encoding genes in marine phage of all types reflects the importance of scavenging nucleic acids for DNA synthesis during phage infection in the often nitrogen- and phosphorous-limited oceanic environments.

### Host genomic islands and a putative P-SS2 integration site

**Fig. 3:**
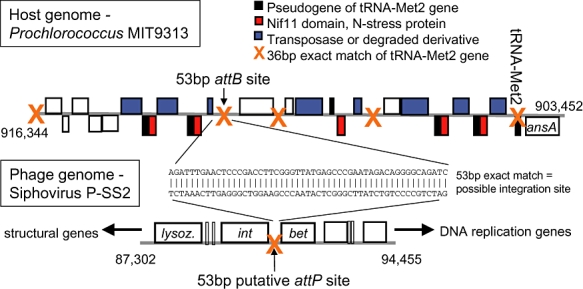
Schematic representation of genome regions surrounding the putative phage (P-SS2) and host (*Prochlorococcus* MIT9313, GenBank ID: NC_005071) integration sites. This site consists of a 53 bp exact match between the phage sequence downstream of its integrase gene at position 90,836–90,888, and the non-coding sequence in the host genome at position 912,261–912,313. This general region of the host genome is a genomic island, and thus hypervariable (see text). Numbers at the genome ends represent the nucleotide position in the respective genomes.

The P-SS2 genome contains three of the four genes that are considered hallmark lysogeny genes in lambda: *int*, *exo* and *bet* (Table 2, Fig. 1B). If capable of integration as a prophage, then two more protein functions must be present as well. First, the function of the fourth hallmark lysogeny gene, excisionase, would need be filled by a host-encoded version as has been observed for other phages and plasmids that use host-encoded site-specific recombinases (Barre and Sherratt, 2002; Huber and Waldor, 2002). Second, a repressor is critical to remain integrated as a prophage to prevent expression of lytic genes that would induce the prophage out of its host genome. While P-SS2 lacks an identifiable repressor, these are small proteins that are highly divergent and often not recognizable even in known functional prophages (e.g. marine *Silicibacter* prophages; Chen *et al.*, 2006). In addition to *int*, *exo* and *bet*, the P-SS2 genome contains a 53 bp intragenic non-protein-coding sequence between *int* and *bet* that exactly matches an intergenic, non-coding region of host *Pro*MIT9313 and includes 36 bp that exactly match a nearby tRNA-Met (Fig. 3). No other matches outside of the 36 bp coding region of the tRNA-Met were found in GenBank, the Global Ocean Survey microbial metagenomes, or the microbial genomes at Microbes Online. Prophages commonly integrate into conserved host genome sites such as tRNA (Campbell, 2003) and tmRNA (Williams, 2002). Thus this exceedingly rare 53 bp match to the non-coding host genome sequence which should be prone to amelioration by neutral mutation and that maintains partial identity to a tRNA in the host genome may represent the phage (*attP*) and host (*attB*) site-specific attachment sites. Near the putative integration site in the host genome are signatures of mobile genetic element activity including eight transposase genes, five pseudo-tRNA-Met genes and five copies of 36 bp of the 53 bp exact match (Fig. 3). However, these mobile genetic elements are likely relics of long-ago transposition events because the transposase genes are variously degraded and lack identifiable inverted-repeat ‘ends’ (see *Experimental procedures*).

**Fig. 4:**
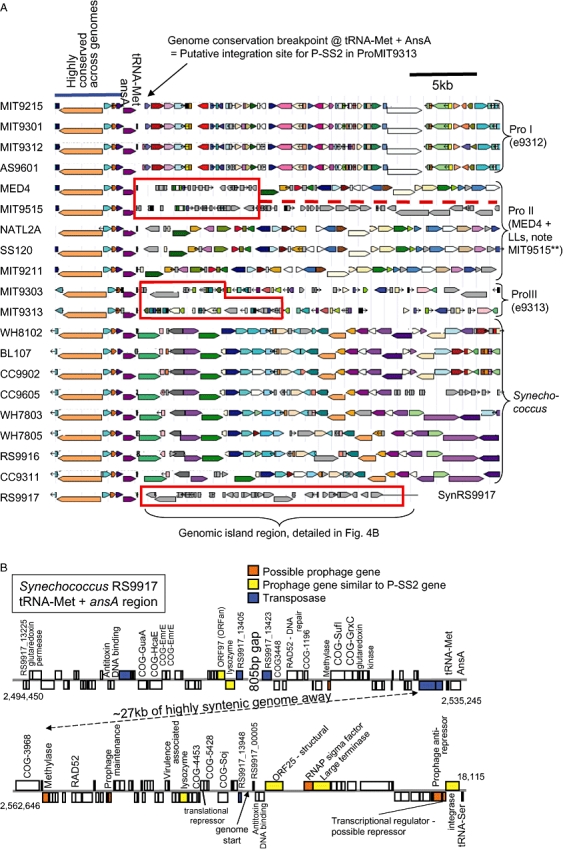
Genome arrangement at the tRNA-Met +*ansA* locus across (A) *Prochlorococcus* and *Synechococcus* genomes, and (B) detailed for *Synechococcus* RS9917. A. Comparative genomics of marine *Prochlorococcus* and S*ynechococcus* at the tRNA-Met +*ansA* locus identified as the putative P-SS2 integration site in *Pro*MIT9313. Across the marine cyanobacteria, this region is highly syntenic with four basic genome patterns observed – denoted as *ProI, ProII, ProIII*, and *Synechococcus* in the figure. However, some strains lack synteny and have hypervariable or ‘genomic islands’ regions, indicated by the red boxes in the figure. MED4 has a small ∼8 kb island with phage high-light inducible genes (this is equivalent to ISL2, Coleman *et al.*, 2006), while MIT9515 has a slightly larger and similar island to MED4s then a region that is a large genome rearrangement (red dashed line) that is syntenic to another region of the MED4 genome (647,805–687,505). The eMIT9313 variability in this region is detailed in Fig. 3. B. The tRNA-Met + AnsA region in *Synechococcus* RS9917 that is homologous to the putative *attB* integration site in *Pro*MIT9313 from Fig. 3. This ∼41 kb ‘island’ region contains four transposases, an antitoxin gene, and two PSS2-like genes – lysozyme and structural protein ORF97. Genomic synteny to all the other marine cyanobacteria then continues for ∼27 kb until reaching a second ∼42 kb ‘island’ that is bounded on the other side by tRNA-Ser, and contains a transposase, as well as numerous prophage-related genes including a possible repressor, antirepressor, prophage maintenance protein, RNAP sigma factor, and four PSS2-like genes – large terminase, integrase, ORF25 structural gene, lysozyme. The COG categories refer to those at Microbes Online.

While future work is required to experimentally prove that this 53 bp match is the integration site for P-SS2 in its host, we chose to examine the available marine *Prochlorococcus* and *Synechococcus* genomes in this tRNA-Met + *ansA* region. Across 21 genomes, this homologous region revealed a complex evolutionary story (Fig. 4A). In each examined host genome, this region is highly conserved right up to the tRNA-Met gene from the *ansA* gene side of this tRNA (blue bar in Fig. 4A). Among some genomes, synteny continues among subgroups of these strains (*ProI, ProII, ProIII, Synechococcus* labels in Fig. 4A). In contrast, a number of genomes have hypervariable ‘genomic island’ regions (*sensu*Coleman *et al.*, 2006) at this tRNA breakpoint (red squares in Fig. 4A). Some of these ‘island’ regions are small, as in the *Prochlorococcus* strains MED4 and MIT9515, and contain numerous *hli* genes, which appear to have been horizontally transferred to these genomes by phages (Lindell *et al.*, 2004). Others are more extensively hypervariable, as in *Pro*MIT9313 (described above, detailed in Fig. 3) and *Syn*RS9917 (detailed in Fig. 4B). The *Syn*RS9917 island region is 41 kb in size and lacks *hli* genes but contains six transposases and two P-SS2-like genes – lysozyme and ORF97 (Fig. 4B). Further, ∼27 kb of highly syntenic genome away is a second genomic island of ∼42 kb, bounded by a tRNA-Ser (Fig. 4B). This island contains another transposase, assorted genes related to prophage induction including maintenance proteins, an antirepressor and a possible repressor, as well as four P-SS2-like genes – large terminase, integrase, RNAP sigma factor, ORF25 structural gene, lysozyme. This is most certainly a relic prophage that shared some similarity with P-SS2.

**Fig. 5:**
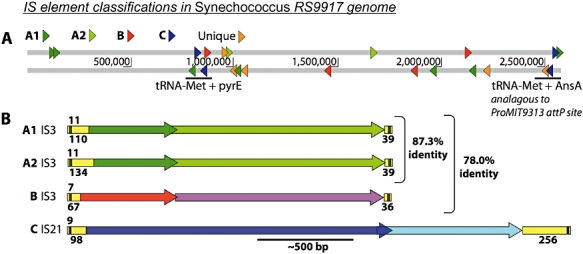
Characterization of insertion sequence (IS) elements in *Synechococcus* RS9917. The 22 transposase genes and surrounding regions (the IS element) revealed four groups of multicopy IS elements, and five unique or degraded IS elements in the SynRS9917 genome. Using the ACLAME database, we classified these IS elements as follows. The two multicopy groups ‘A1’ and ‘A2’ are IS3-like mobile elements and have identical inverted repeats, identical lengths and > 87% sequence identity. IS group ‘B’, is also IS3-like element but has a shorter inverted repeat. IS group ‘C’ is longer and most similar to IS21-like elements. A. Location and orientation of IS elements, represented by coloured arrows, in relation to the proposed P-SS2-like phage integration sites (regions represented by black bars). B. Diagrams of IS elements including size of inverted repeat (size in bp indicated above the 5′-end of the yellow box), position and orientation of ORFs, and size of flanking non-coding regions (shown in yellow, with size in bp indicated below the yellow box).

**Fig. 6:**
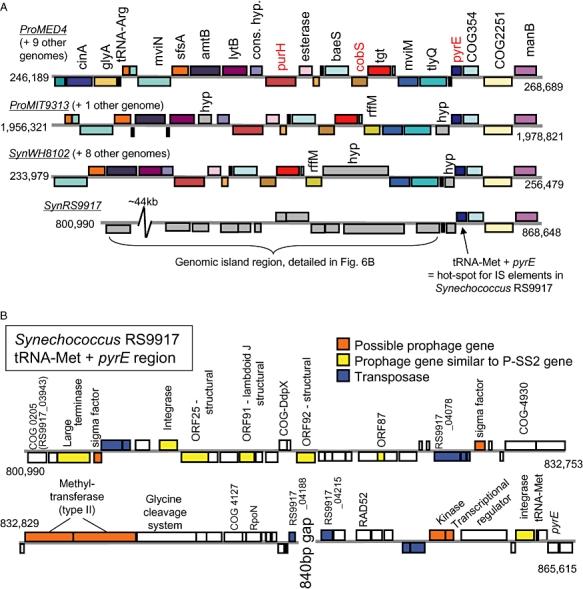
Genomic arrangement of the tRNA-Met +*pyrE* site in *Synechococcus* RS9917 identified as a secondary hot-spot for insertion sequence elements. A. Schematic of the highly syntenic tRNA-Met +*pyrE* region from representative *Prochlorococcus* and *Synechococcus* genomes. Minor insertions in *Pro*MIT9303 and *Pro*MIT9313 (*rffM* insertion, small hypothetical ORFs) and the marine *Synechococcus* (*rffM*+ large hypothetical ORF) are the only deviations from complete synteny in this region, except for the genomic island detailed for *Synechococcus* RS9917 (see Fig. 6B). Gene names are listed for the top genome only, and homologues across the genomes are similarly coloured. Red gene names have been previously observed in myovirus cyanophage genomes (Sullivan *et al.*, 2005). Nine other genomes are similar to the *Prochlorococcus* MED4 arrangement, one other for the MIT9313 arrangement, and eight other for the *Synechococcus* WH8102 arrangement (details in Table S2). B. In contrast to the genome conservation observed in other *Prochlorococcus* and *Synechococcus* genomes, *Syn*RS9917 contains a ∼65 kb genomic island region that contains nine transposases, seven P-SS2-like genes and seven phage-like genes. This is the most intact prophage in any marine *Prochlorococcus* or *Synechococcus* genome, but it is still significantly degraded.

In contrast to the *Pro*MIT9313 transposases described above, the bulk of the *Syn*RS9917 transposase genes appear as intact composite IS element with identifiable ‘ends’ (Fig. 5). The presence of two RAD52 family proteins (Fig. 4B) suggests the need for double-stranded DNA repair as if this region were under heightened ‘attack’ from IS elements. Notably, a second hot-spot of IS elements occurs at *pyrE* in the *Syn*RS9917 genome. This region contains another tRNA-Met and RAD52 family protein, and is syntenic across all host genomes examined except for a 65 kb genomic island in *Syn*RS9917 (Fig. 6A). This *Syn*RS9917 island contains 13 phage-related genes (seven of which are similar to P-SS2 genes, Fig. 6B) and, while clearly incomplete, represents the most intact marine cyanobacterial prophage observed to date. Notably, among the 21 *Prochlorococcus* and *Synechococcus* host strains available for testing at the time, P-SS2 infected only its original host used for isolation, *Pro*MIT9313 (note: *Syn*RS9917 was not available for testing, Sullivan *et al.*, 2003).

### Insertion sequence-mediated genome evolution in *Pro*MIT9313

While the tRNA-Met + AnsA genomic island in *Pro*MIT9313 described above contains variously degraded IS elements, this region may not simply be a ‘graveyard’ of degraded genes and pseudogenes. All five transposases are bordered by paralogous genes with sequence similarity to a nif11-domain (Fig. 3) that suggests a nitrogen stress-related function based upon annotation alone. Indeed, nitrogen stress, and not phosphate stress, alters the gene expression of four of five of these nif11-domain-containing genes (Martiny *et al.*, 2006; Tolonen *et al.*, 2006). Across diverse bacterial genomes, IS elements often alter expression of neighbouring genes using outward facing promoters (Mahillon and Chandler, 1998). Insertion sequence elements are also capable of transporting ‘cargo’ genes around genomes (Poirel *et al.*, 2005; Toleman *et al.*, 2006; Bartosik *et al.*, 2008), often with greater transposition efficiency than wild-type elements (Bartosik *et al.*, 2008) and lead to fitness gains under experimental evolutionary conditions (Schneider and Lenski, 2004) and in the generation of reduced symbiont genomes (Moran and Plague, 2004; Plague *et al.*, 2008). Thus we hypothesize that these *Pro*MIT9313 IS elements may both move around (as ‘cargo’) and regulate (via outward-facing promoters) these proximal nitrogen stress genes thereby contributing to host niche differentiation.

Further, this region is coincident with the putative P-SS2 siphovirus integration site, suggesting two layers of genome evolution. Insertion sequence elements may mediate *intra*genomic innovation by bringing genes to this region as an evolutionary ‘sandpit’ where selection challenges new combinations of alleles and genes as appears to be the case for the nif11-domain genes above. Then, if indeed P-SS2 is capable of integration at this site, the phage could obtain such IS-mediated genetic innovation through aberrant excision and distribute it to other host genomes (*inter*genomic innovation) via new infections. If this were occurring, then one might expect to occasionally observe IS elements from such evolutionary action in phage genomes. Indeed, in spite of very few environmental phage genomes available, IS transposase genes have been observed in marine phages (e.g. vibriophage VHML; Oakey *et al.*, 2002; Chibani-Chennoffi *et al.*, 2004) and freshwater cyanophages (Ma-LMM01 has 3 transposases; Yoshida *et al.*, 2008). Further, IS elements are known to facilitate the spread and expression of genes enabling antibacterial resistance and degradation of toxic compounds (Berg and Howe, 1989; Bushman, 2002; Nojiri *et al.*, 2004). Perhaps here nitrogen stress response is similarly tied to IS-mediated evolution in marine cyanobacteria, which are often N-limited.

### The acquisition of ‘host genes’ into the cyanophage genome pool

While ‘host genes’ or ‘auxiliary metabolic genes’ (AMGs) are commonly observed in cyanophage genomes (reviewed in Breitbart *et al.*, 2007), it remains unclear how they are obtained by cyanophages. Notably, a second IS-element hot-spot in *Syn*RS9917 is again located at a tRNA-Met which in 21 other *Prochlorococcus* and *Synechococcus* genomes is proximal to *pyrE*, *cobS* and *purH* (Fig. 6A) – three genes that are found in lytic myovirus cyanophage genomes (Syn9, P-SSM2 and P-SSM4; Sullivan *et al.*, 2005, Weigele *et al.*, 2007). In contrast, this region in the *Syn*RS9917 genome has significant evidence of past prophage-integration activity including 13 phage-related genes, seven of which share sequence similarity with P-SS2 genes (Fig. 6B). While this prophage is clearly a relic (nine transposases, missing many genes), could such a prophage have introduced these three ‘host genes’ or AMGs into the phage genome pool? Induced prophages can improperly excise from the host genome and mispackage up to 10% of the host genome proximal to the integration site in place of part or all of the phage genome (Calendar, 1988). Thus the remnant *Syn*RS9917 prophage(s) may have initially obtained such AMGs proximal to this site-specific integration site. These genes could then have been disseminated to super-infecting lytic cyanophages through recombination. Such prophage-to-lytic-phage recombination events are thought to be among the most probable means of spreading new genetic material through the phage genome pool as has been observed in *Streptococcus thermophilus* and *Lactococcal* phages (Brussow and Desiere, 2001), mycobacteriophages (Pedulla *et al.*, 2003), and more generally the siphoviruses (Hendrix *et al.*, 1999).

### Prevalence of P-SS2-like siphoviruses in the surface oceans

Given that siphoviruses have rarely been isolated in studies using a diversity of marine cyanobacterial hosts to isolate phage from seawater (Waterbury and Valois, 1993; Suttle and Chan, 1994; Lu *et al.*, 2001; Marston and Sallee, 2003, Sullivan *et al.*, 2003), one wonders whether this was a function of isolation procedures or whether they occur in relatively low abundances in the wild. To begin to address this question, we used the P-SS2 genome to ‘recruit’ homologous fragments from the microbial fraction Global Ocean Survey surface ocean metagenomes (see *Experimental procedures*). There were only seven GOS reads with a best hit to the P-SS2 genome (Table S3), and the alignment lengths of these hits were short, ranging from 47 to 242 bp. Homologues of the *Pro*MIT9313 genome are also rare in the GOS data set (< 0.35% of the total hits in any given site, data not shown), which is not surprising as these LL-adapted *Prochlorococcus* cells are not abundant in surface waters (Johnson and Zinser *et al.*, 2006). Thus this limited analysis suggests that siphoviruses similar to the one used in this study are not abundant in surface ocean waters, but may be in undersampled lower euphotic zone waters.

## Conclusions

The ocean cyanobacterial siphovirus P-SS2 contains a large genome that is significantly divergent from the siphovirus genomes sequenced to date, so much so that even structural proteins required experimental validation to annotate. This contrasts with the classically lytic phages (e.g. T4-like myoviruses and T7-like podoviruses) which exemplify a cohesive genomic architecture ranging from non-marine coliphages (Miller *et al.*, 2003; Nolan *et al.*, 2006) to marine representatives of roseophages (Rohwer *et al.*, 2000), vibriophages (Miller *et al.*, 2003) and cyanophages (Chen and Lu, 2002; Mann *et al.*, 2005; Sullivan *et al.*, 2005; Pope *et al.*, 2007; Weigele *et al.*, 2007). The siphoviruses, however, are thought to be prone to extensive genetic module ‘swapping’ through intensive recombination (Hendrix *et al.*, 1999; Juhala *et al.*, 2000; Brussow and Desiere, 2001; Proux *et al.*, 2002; Pedulla *et al.*, 2003), thus the divergence observed is not surprising. This intense mosaicism causes siphoviruses to display web-like phylogenies (Brussow and Desiere, 2001) and to represent the most taxonomically challenging phage group (Hendrix *et al.*, 1999; Edwards and Rohwer, 2005; Lawrence *et al.*, 2002; Proux *et al.*, 2002). Beyond the P-SS2 genome, exploration of a putative phage integration site in the host genome revealed extensive genomic islands in the host and IS elements among some ocean *Prochlorococcus* and *Synechococcus* genomes at this location. Particularly striking are the IS element hot-spots in *Syn*RS9917 where the comingling components of the cyanobacterial ‘mobilome’ revealed evidence of prophages under IS element attack, as well as a possible mechanism for phage-captured ‘host ‘AMGs central to cyanophage biology (reviewed in Breitbart *et al.*, 2007).

## Experimental procedures

### Isolation of the phage and preparation for genomic sequencing

The siphovirus P-SS2 was isolated from Atlantic Ocean slope waters (38°10′N, 73°09′W) collected on 17 September 2001 on the R/V Endeavor cruise number 360. The sampled water was from 83 m depth with a salinity 36.6 ppt and temperature 20.8°C. This water was 0.2 μm filtered and stored at 4°C until it was used directly in a plaque assay with *Prochlorococcus* strain MIT9313 as a host on 12 December 2001. A large, well-resolved plaque was picked from the lawn of host cells on 29 December 2001, plaque purified two more times and stored as a lysate of a P-SS2 clonal stock isolate.

P-SS2 was prepared for sequencing as previously described (Lindell *et al.*, 2004). Briefly, phage particles were concentrated from large volume (2 l) lysates using polyethylene glycol. Concentrated DNA-containing phage particles were purified from other material in phage lysates using a density caesium chloride gradient. Purified phage particles were broken open (SDS/proteinase K), and DNA was extracted (phenol:chloroform) and precipitated (ethanol) yielding small amounts of DNA (< 1 ng). A custom 1–2 kb insert linker-amplified shotgun library was constructed by Lucigen (Middletown, WI, USA) as described previously (Breitbart *et al.*, 2002). Additional larger insert (3–8 kb) clone libraries were constructed from genomic DNA by the Department of Energy (Joint Genome Institute, Walnut Creek, CA, USA) using a similar protocol to provide larger scaffolds during assembly. Inserts were sequenced by the Department of Energy Joint Genome Institute from all clone libraries and used for initial assembly of these phage genomes. The Stanford Human Genome Center Finishing Group (Palo Alto, CA, USA) closed the genomes using primer walking.

### Genome annotation

Gene identification and characterization was done as in Sullivan and colleagues (2005). Briefly, protein coding genes were predicted using GeneMark and manual curation. Translated ORFs were compared with known proteins in the non-redundant GenBank and in the KEGG databases using the blastp program. Where blastp*e*-values were high (> 0.001) or no sequence similarity was observed, ORF annotation was aided by the use of PSI-blast, gene size, domain conservation, and/or synteny (gene order). Identification of tRNA genes was done using tRNAscan-SE. Additionally, rho-independent transcription terminators were identified with TransTermHP (Kingsford *et al.*, 2007) using default parameters. All terminators had a confidence score > 80% with an energy score of < −15 and a tail score of < −6. Bacterial σ^70^ promoters were predicted using BPROM (Softberry, Mount Kisco, NY, USA) using default parameters. All intergenic promoters with a linear discriminant function > 3.5 were considered candidate promoters. Inverted repeats of IS elements were found using the Palindrome program in the EMBOSS software suite (Rice *et al.*, 2000). Approximately 200 bp upstream and downstream of each putative IS element was fed into Palindrome and the output of many inverted repeats identified were screened manually to identify those that were exact matches and at least 7 bp long. Insertion sequence elements were classified using the ACLAME database blast tool (Leplae *et al.*, 2004). Genome visualizations were done in Artemis (Rutherford *et al.*, 2000), while comparative genomics analyses were greatly aided by the tools available at MicrobesOnline (http://www.microbesonline.org). For figure labels where genes are denoted as ‘prophage’ or ‘cyanobacterial’, these assignments were made using NCBI taxonomy lineages of the top 5 blast hits. In all cases where these are denoted, all five top hits were of one of these two organismal types, cyanobacteria or known temperate phages or integrated prophages, with *e*-values < 0.001. The resulting genome sequence is deposited under GenBank accession #GQ334450.

### Ocean microbial metagenomic analyses

To determine whether P-SS2 occurred in the wild, we queried the Global Ocean Survey (GOS; Rusch *et al.*, 2007) microbial surface ocean water metagenomes. We created a database of all sequenced marine isolates, including Gordon and Betty Moore Foundation Marine Microbial Initiative genomes, NCBI marine isolates, and cyanophage available from the GenBank and CAMERA databases as of November 2008. Environmental metagenomic reads were blasted (blastall -p blastn -e 1e-5 -z 25000000000 -m 7 -a 4 -F ‘m L’ -X 150 -U T) against this database. Best hits to each GOS read were retrieved and filtered by alignment length. Reads with best hits to P-SS2 and *Pro*MIT9313 (GenBank ID: NC_005071) are the focus of this study.

### Large terminase (TerL) and group 3 sigma factor protein phylogenies

Protein alignments were generated using the Promals web server (Pei and Grishin, 2007; Pei *et al.*, 2007) using default parameters and manually edited as needed. Amino acid distance trees were constructed using the paup*4.0b10 software. Neighbour joining was used to reconstruct distance trees using minimum evolution as the objective function and uncorrected distances. Amino acid maximum likelihood trees were inferred using the CIPRES web portal RAxML rapid bootstrapping and ML search (Stamatakis, 2006; Stamatakis *et al.*, 2008) assuming the James-Taylor Thornton model of substitution using empirical base frequencies and estimating the proportion of invariable sites from the data.

### Virion structural proteomics

Briefly, the samples were incubated in a denaturing solution of 8 M Urea/1% SDS/100 mM ammonium bicarbonate/10 mM DTT pH 8.5 at 37°C for 1 h. Next, the samples were alkylated for 1 h by the addition of iodoacetamide to a final concentration of 40 mM and then quenched with 2 M DTT. Following the addition of 4× LDS loading buffer (Invitrogen), each sample was centrifuged at 14 000 r.p.m. for 5 min at room temperature, and each sample was fractionated on a NuPAGE 10% Bis-Tris 10 lane gel (Invitrogen) for 2.5 h at 125 V, 50 mA and 8 W. Gels were shrunk overnight by the addition of 50% ethanol and 7% acetic acid, and then allowed to swell for 1 h by the addition of deionized water. Gels were stained with SimplyBlue Safe Stain (Invitrogen) for 2–4 h, imaged, and sliced horizontally into fragments of equal size based on the molecular weight markers.

In-gel digestion was performed after destaining and rinsing the gel sections with two washes of 50% ethanol and 7% acetic acid, followed by two alternating washes with 50 mM ammonium bicarbonate and acetonitrile. After removal of the last acetonitrile wash, 100 μL of sequencing grade trypsin (Promega) was added to each gel slice at a concentration of 6.6 ng μl^−1^ in 50 mM ammonium bicarbonate/10% acetonitrile. The gel slices were allowed to swell for 30 min on ice, after which the tubes were incubated at 37°C for 24 h. Peptides were extracted with one wash of 100 μl of 50 mM ammonium bicarbonate/10% acetonitrile and one wash of 100 μl of 50% acetonitrile/0.1% formic acid. The extracts were pooled and frozen at −80°C, lyophilized to dryness and redissolved in 40 μl of 5% acetonitrile, 0.1% formic acid.

Samples were then loaded into a 96-well plate (AbGene) for mass spectrometry analysis on a Thermo Fisher Scientific LTQ-FT. For each run, 10 μl of each reconstituted sample was injected with a Famos Autosampler, and the separation was performed on a 75 mM × 20 cm column packed with C18 Magic media (Michrom Biosciences) running at 250 nl min^−1^ provided from a Surveyor MS pump with a flow splitter with a gradient of 5–60% water 0.1% formic acid, acetonitrile 0.1% formic acid over the course of 120 min (150 min total run). Between each set of samples, standards from a mixture of 5 angiotensin peptides (Michrom Biosciences) were run for 2.5 h to ascertain column performance and observe any potential carryover that might have occurred. The LTQ-FT was run in a top five configuration with one MS 200K resolution full scan and five MS/MS scans. Dynamic exclusion was set to 1 with a limit of 180 s with early expiration set to 2 full scans.

Peptide identifications were made using SEQUEST (ThermoFisher Scientific) through the Bioworks Browser 3.3. The data were searched with a 10 ppm window on the MS precursor with 0.5 Dalton on the fragment ions with no enzyme specificity. A reverse database strategy (Elias and Gygi, 2007) was employed with a six frame translation of the genomic sequence reversed and concatenated with the forward sequences supplemented with common contaminates and filtered to obtain a false discovery rate of less than or equal to 1%. Peptides passing the filters were mapped back onto the genome and compared with predicted ORFs.

## Supporting information

**Fig. S1.** Weblogo representation of the consensus promoter sequences predicted across the siphovirus P-SS2 genome. Genomic locations of the predicted promoter sequence locations are presented in Suppl. Table 1 (along with predicted terminators).

**Fig. S2.** Phylogenetic relationships of group 3 sigma factors among phages and microbes. In contrast to group 1 sigma factors which are universal among microbes, these group 3 sigma factor transcriptional regulatory proteins are uncommon among microbes. This is particularly notable among the marine *Prochlorococcus* where they are only found in *Pro*MIT9313 and *Pro*MIT9303. Tree details are as in the Fig. 2 legend, and methods, while in-figure table contains taxa names.

**Fig. S3.** %G+C plot of the siphovirus P-SS2 genome. The black line indicates a sliding base-pair (100 bp) window of %G+C along the genome, while the red line indicates 2.5 times the standard deviation. Notably the major deviation from the genome average (highlighted with the grey box) is where the anomalous tail fibre protein of putative lateral gene transfer origin.

**Table S1.** Genomic locations of predicted promoters and terminators.

**Table S2.** Details of genomic regions sharing synteny with representative genomes presented in Fig. 6A.

**Table S3.** Environmental sequence reads from the Global Ocean Survey (Rusch *et al.*, 2007) that were best hits to the P-SS2 genome.
