# Supplementary material for: The genome and structural proteome of an ocean siphovirus: a new window into the cyanobacterial ‘mobilome’
Source: Environ Microbiol. 2009 Nov;11(11):2935–51. doi: 10.1111/j.1462-2920.2009.02081.x (PMC2784084; doi:10.1111/j.1462-2920.2009.02081.x)
Supplement: Supplementary file 1 [file emi0011-2935-SD1.ppt]

## Slide 1
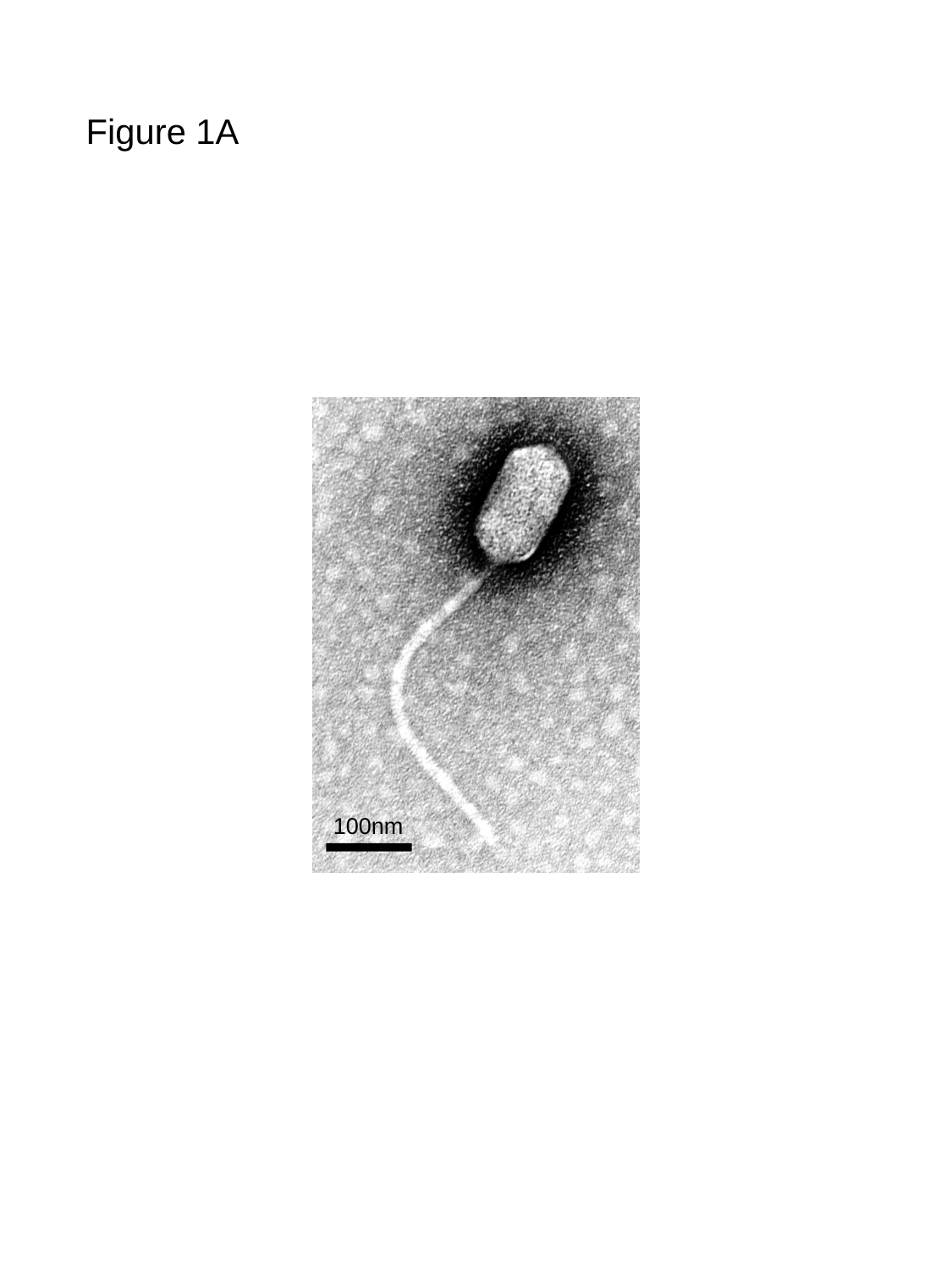

# Figure 1A
100nm

## Slide 2
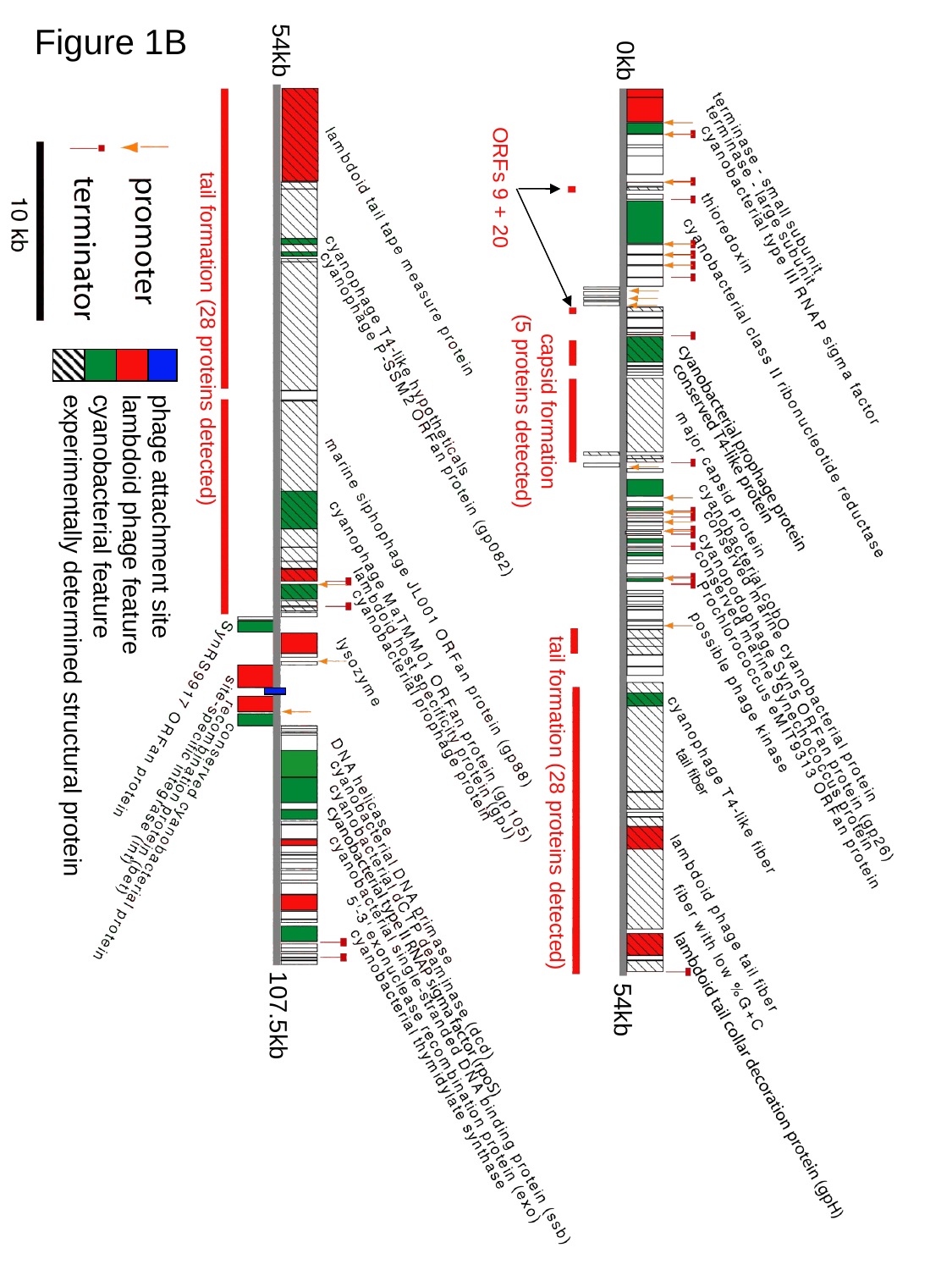

# Figure 1B
54kb
0kb
ORFs 9 + 20
tail formation (28 proteins detected)
capsid formation
(5 proteins detected)
phage attachment site
lambdoid phage feature
cyanobacterial feature
experimentally determined structural protein
tail formation (28 proteins detected)
54kb
107.5kb

## Slide 3
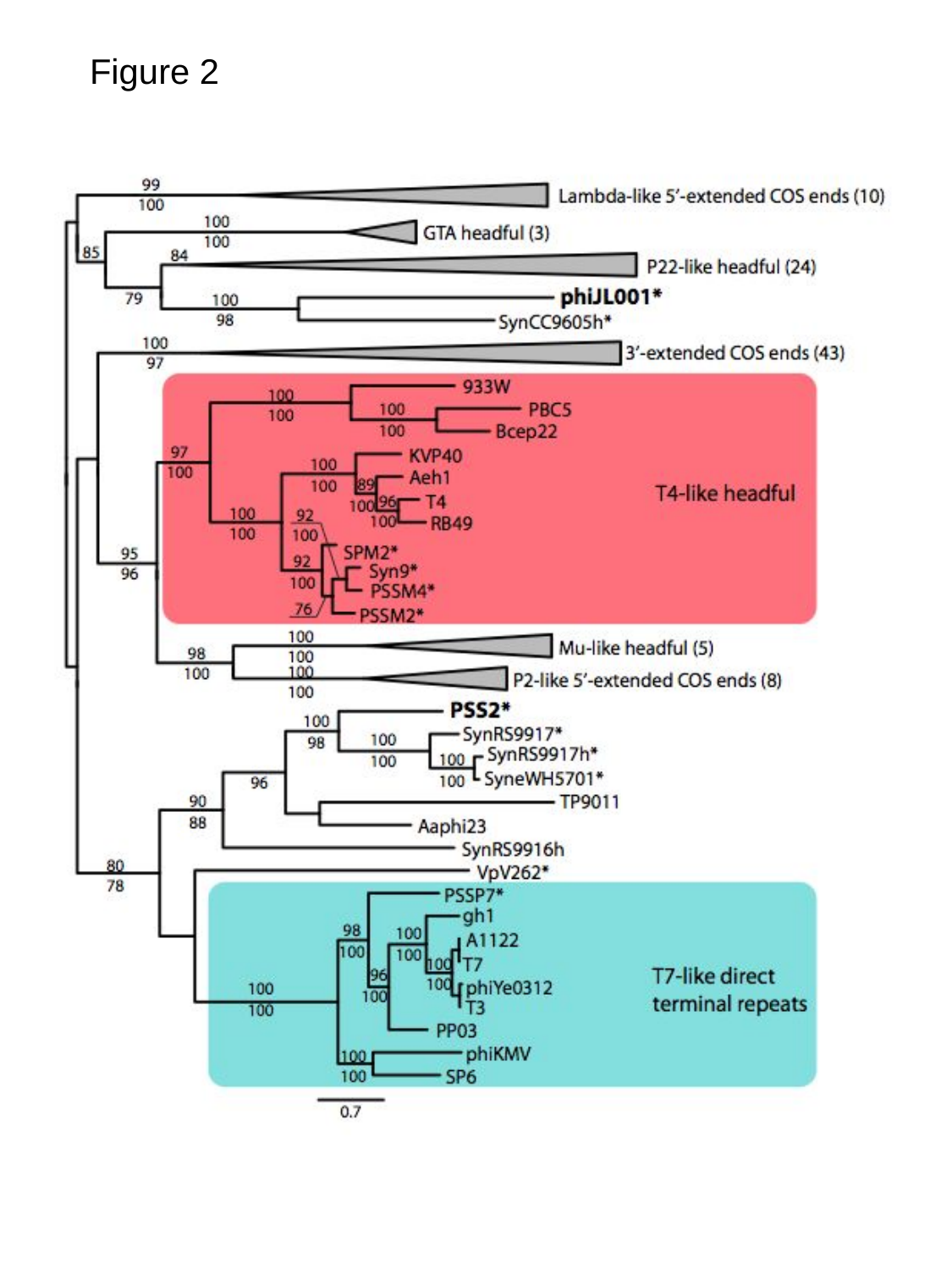

# Figure 2

## Slide 4
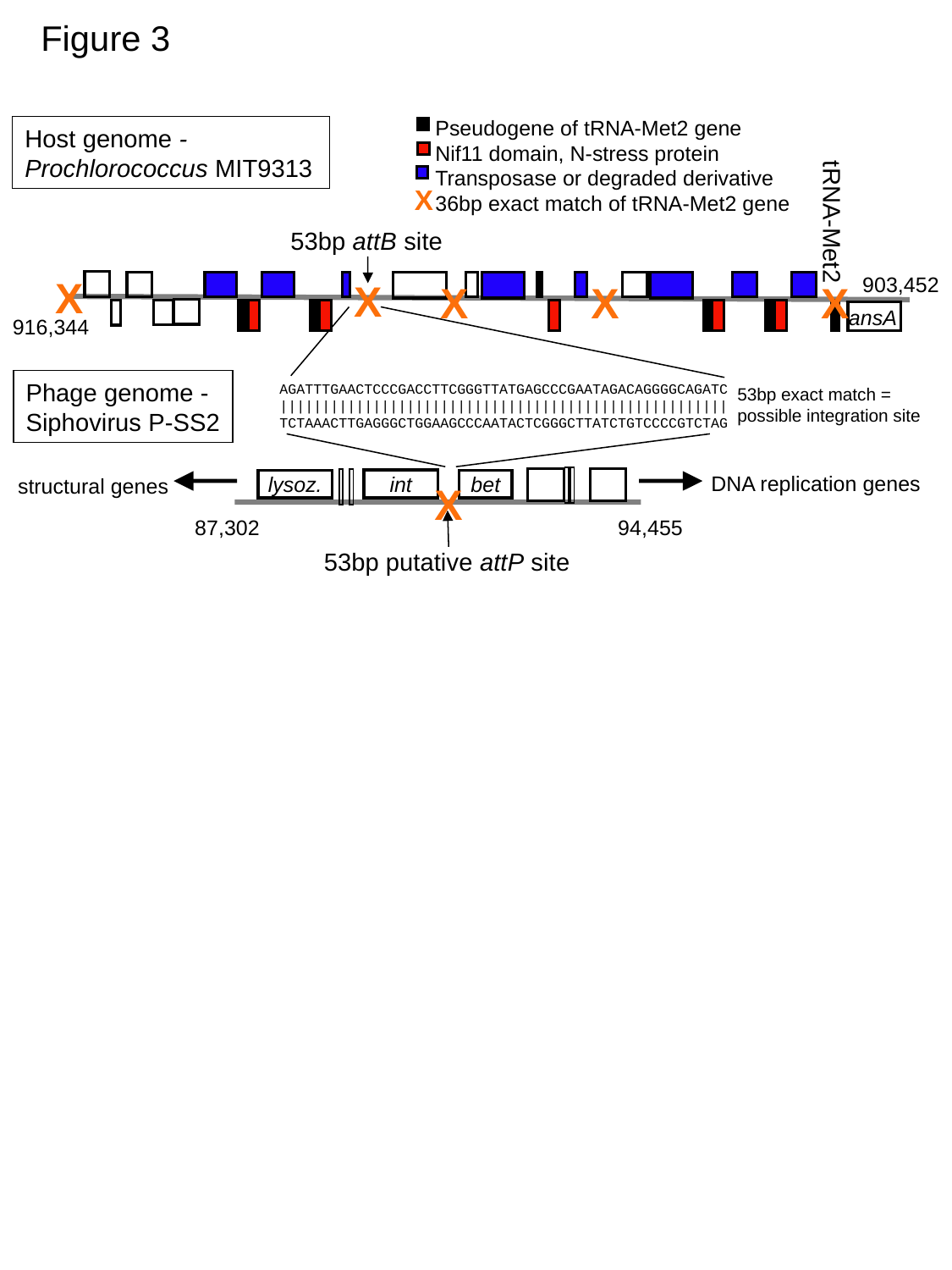

# Figure 3
Pseudogene of tRNA-Met2 gene
Nif11 domain, N-stress protein
Transposase or degraded derivative
36bp exact match of tRNA-Met2 gene
Host genome -
Prochlorococcus MIT9313
X
tRNA-Met2
53bp attB site
X
903,452
X
X
X
X
ansA
916,344
Phage genome -
Siphovirus P-SS2
AGATTTGAACTCCCGACCTTCGGGTTATGAGCCCGAATAGACAGGGGCAGATC
|||||||||||||||||||||||||||||||||||||||||||||||||||||
TCTAAACTTGAGGGCTGGAAGCCCAATACTCGGGCTTATCTGTCCCCGTCTAG
53bp exact match =
possible integration site
DNA replication genes
structural genes
int
lysoz.
bet
X
87,302
94,455
53bp putative attP site

## Slide 5
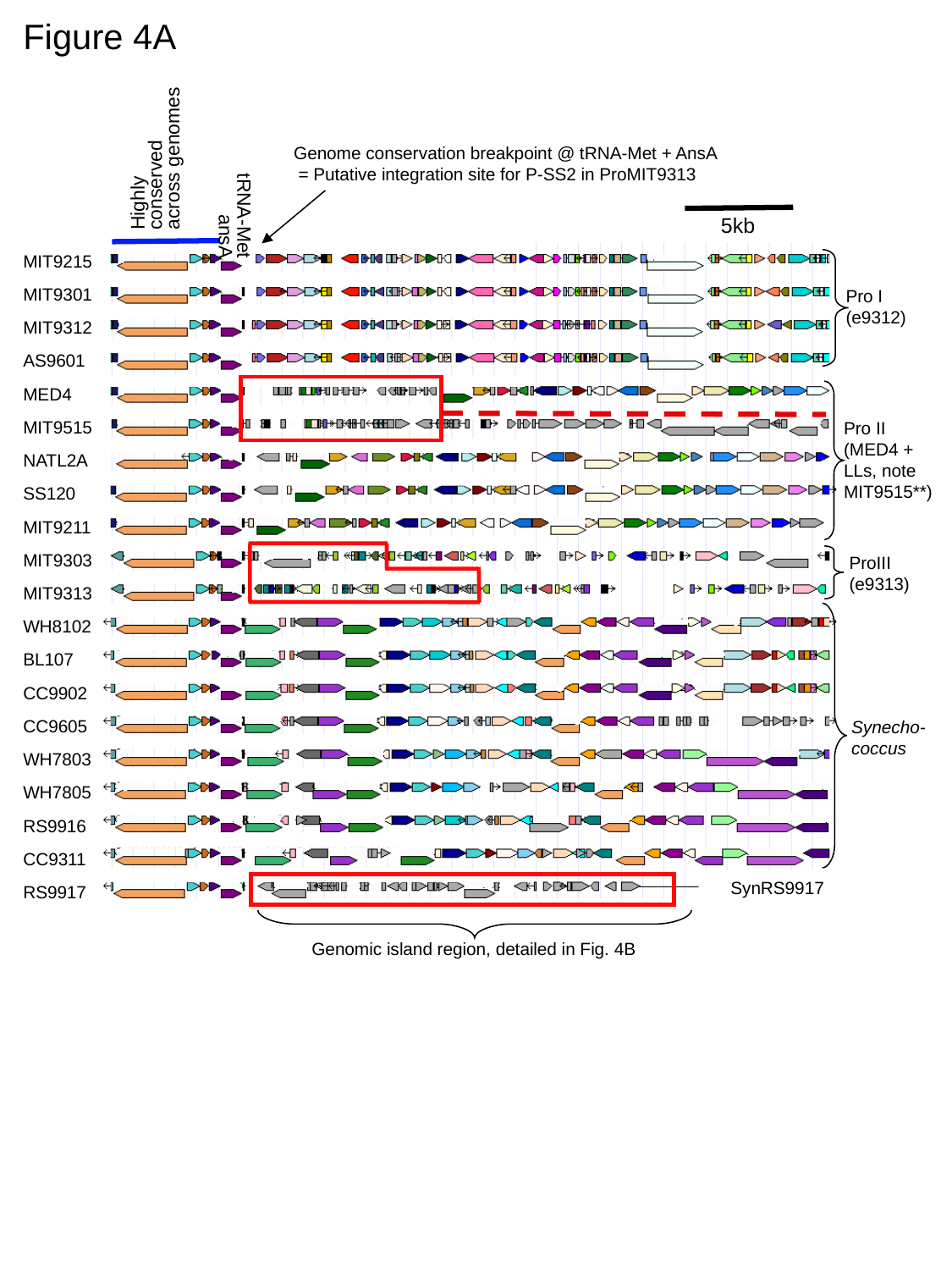

# Figure 4A
Highly
conserved
across genomes
Genome conservation breakpoint @ tRNA-Met + AnsA
 = Putative integration site for P-SS2 in ProMIT9313
tRNA-Met
ansA
5kb
MIT9215
MIT9301
MIT9312
AS9601
MED4
MIT9515
NATL2A
SS120
MIT9211
MIT9303
MIT9313
WH8102
BL107
CC9902
CC9605
WH7803
WH7805
RS9916
CC9311
RS9917
Pro I
(e9312)
Pro II
(MED4 +
LLs, note
MIT9515**)
ProIII
(e9313)
Synecho-
coccus
 SynRS9917
Genomic island region, detailed in Fig. 4B

## Slide 6
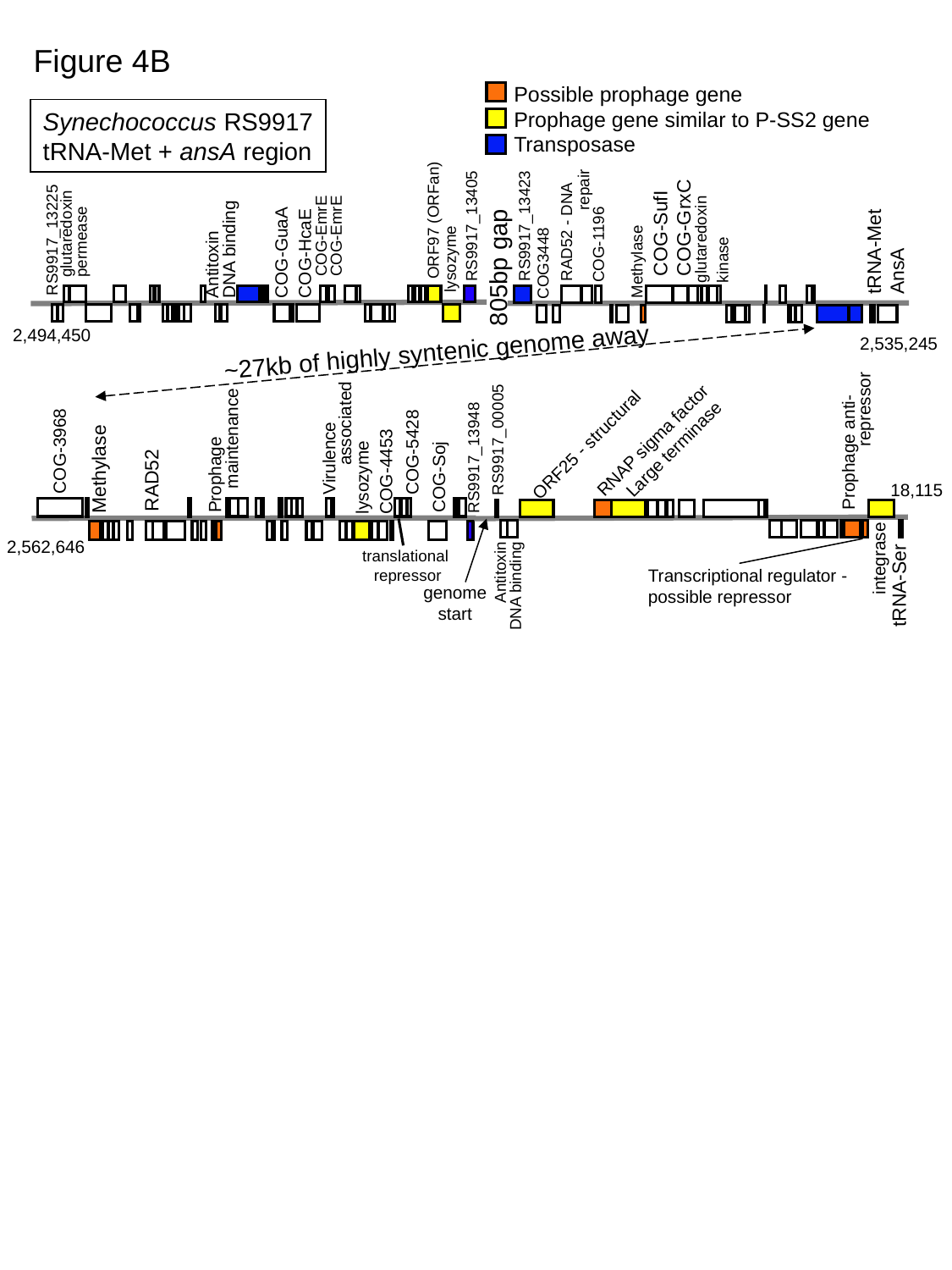

# Figure 4B
Possible prophage gene
Prophage gene similar to P-SS2 gene
Transposase
Synechococcus RS9917
tRNA-Met + ansA region
COG-SufI
COG-GrxC
RAD52 - DNA
 repair
 ORF97 (ORFan)
lysozyme
RS9917_13405
RS9917_13423
glutaredoxin
permease
glutaredoxin
kinase
COG-EmrE
COG-EmrE
tRNA-Met
AnsA
COG-GuaA
COG-HcaE
Antitoxin
DNA binding
RS9917_13225
COG-1196
805bp gap
Methylase
COG3448
2,494,450
2,535,245
~27kb of highly syntenic genome away
Virulence
 associated
Prophage anti-
 repressor
RS9917_00005
RNAP sigma factor
Prophage
 maintenance
ORF25 - structural
Large terminase
COG-3968
COG-5428
RS9917_13948
lysozyme
COG-4453
Methylase
COG-Soj
RAD52
18,115
2,562,646
translational
repressor
integrase
Transcriptional regulator -
possible repressor
Antitoxin
DNA binding
tRNA-Ser
genome
start

## Slide 7
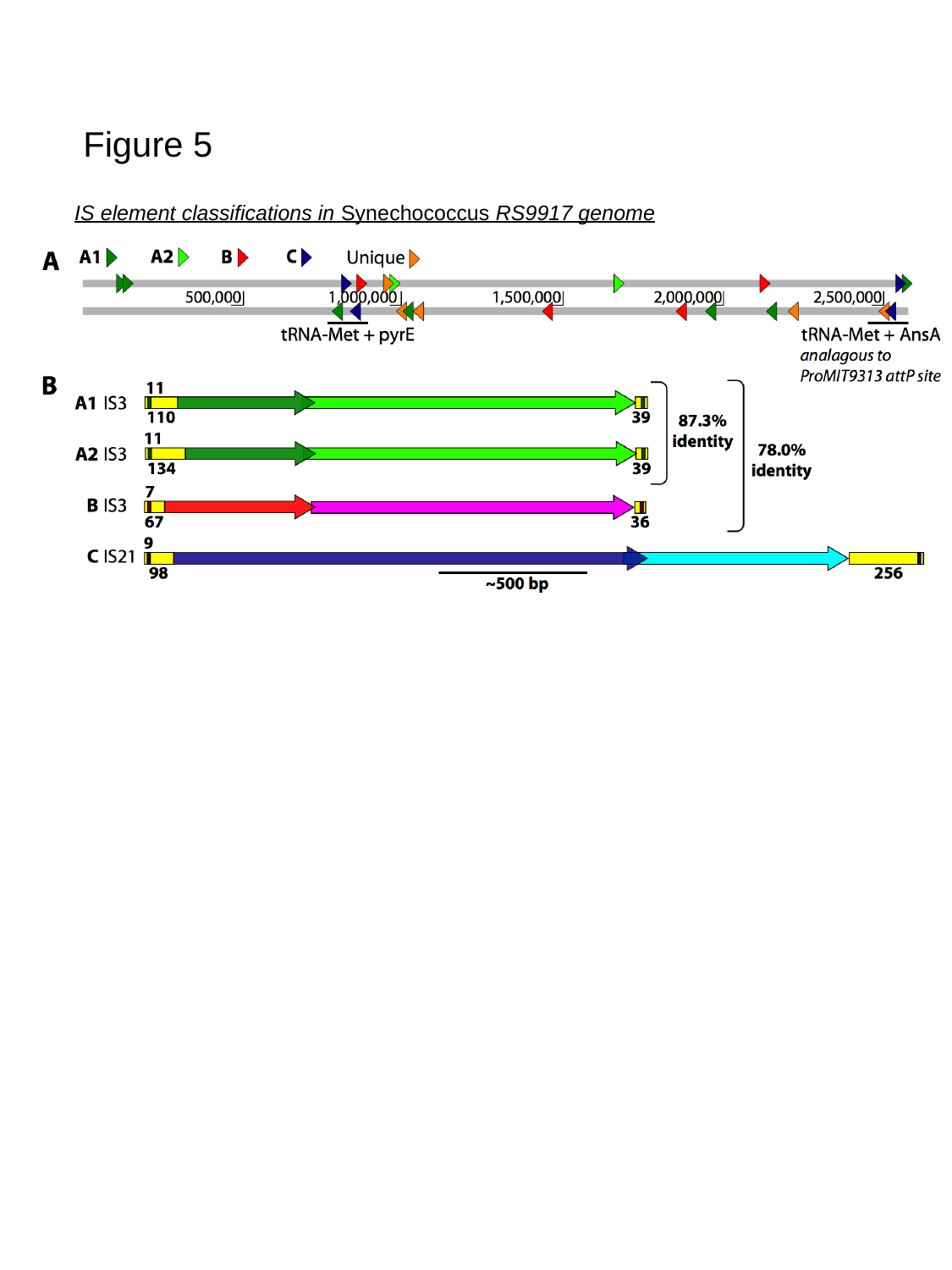

# Figure 5
IS element classifications in Synechococcus RS9917 genome

## Slide 8
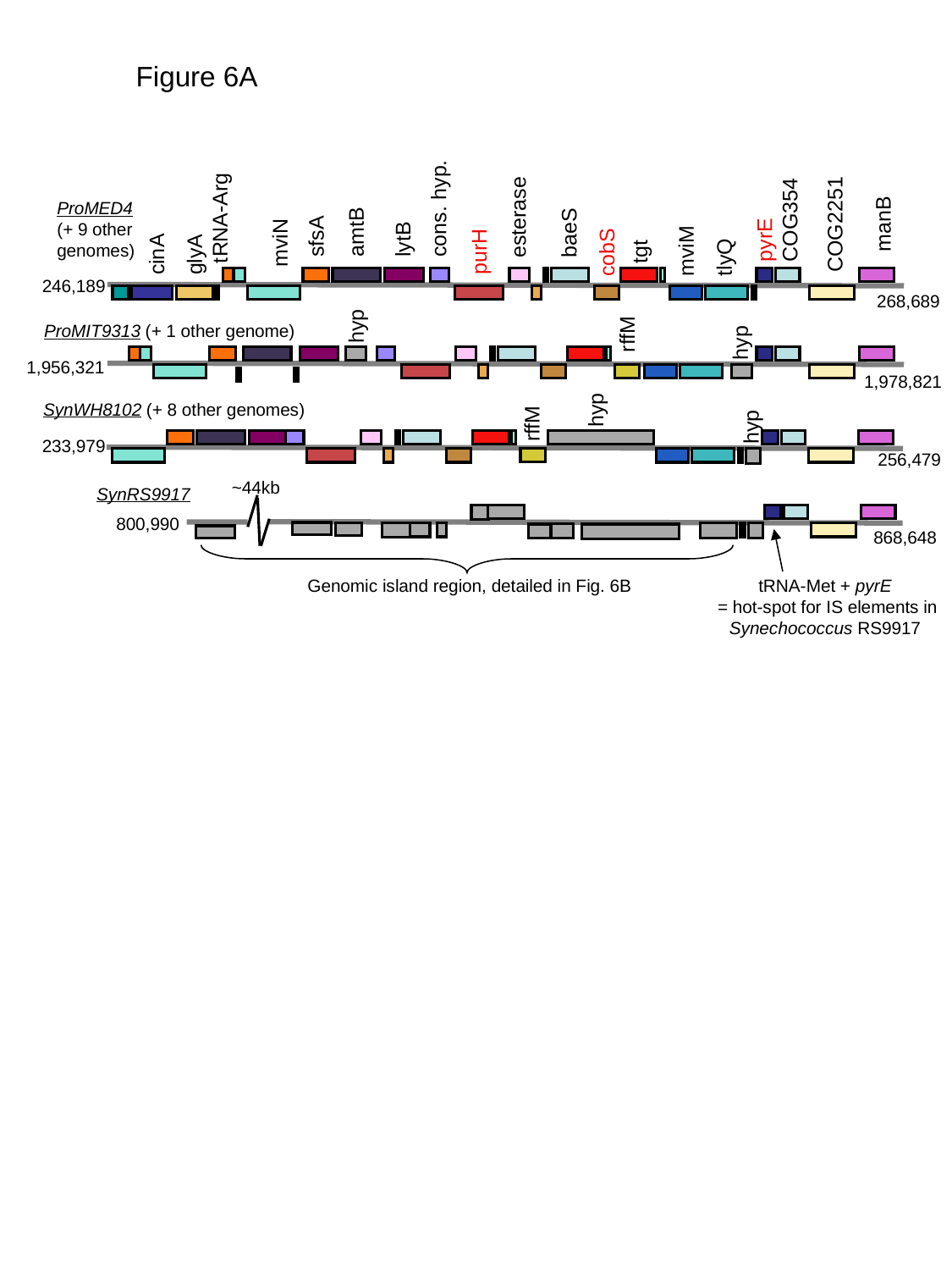

# Figure 6A
sfsA
amtB
lytB
cons. hyp.
esterase
baeS
cinA
glyA
 tRNA-Arg
pyrE
COG354
ProMED4
(+ 9 other
genomes)
manB
COG2251
mviM
tlyQ
mviN
tgt
purH
cobS
246,189
268,689
hyp
ProMIT9313 (+ 1 other genome)
rffM
hyp
1,956,321
1,978,821
hyp
SynWH8102 (+ 8 other genomes)
rffM
hyp
233,979
256,479
~44kb
SynRS9917
800,990
868,648
Genomic island region, detailed in Fig. 6B
tRNA-Met + pyrE
 = hot-spot for IS elements in Synechococcus RS9917

## Slide 9
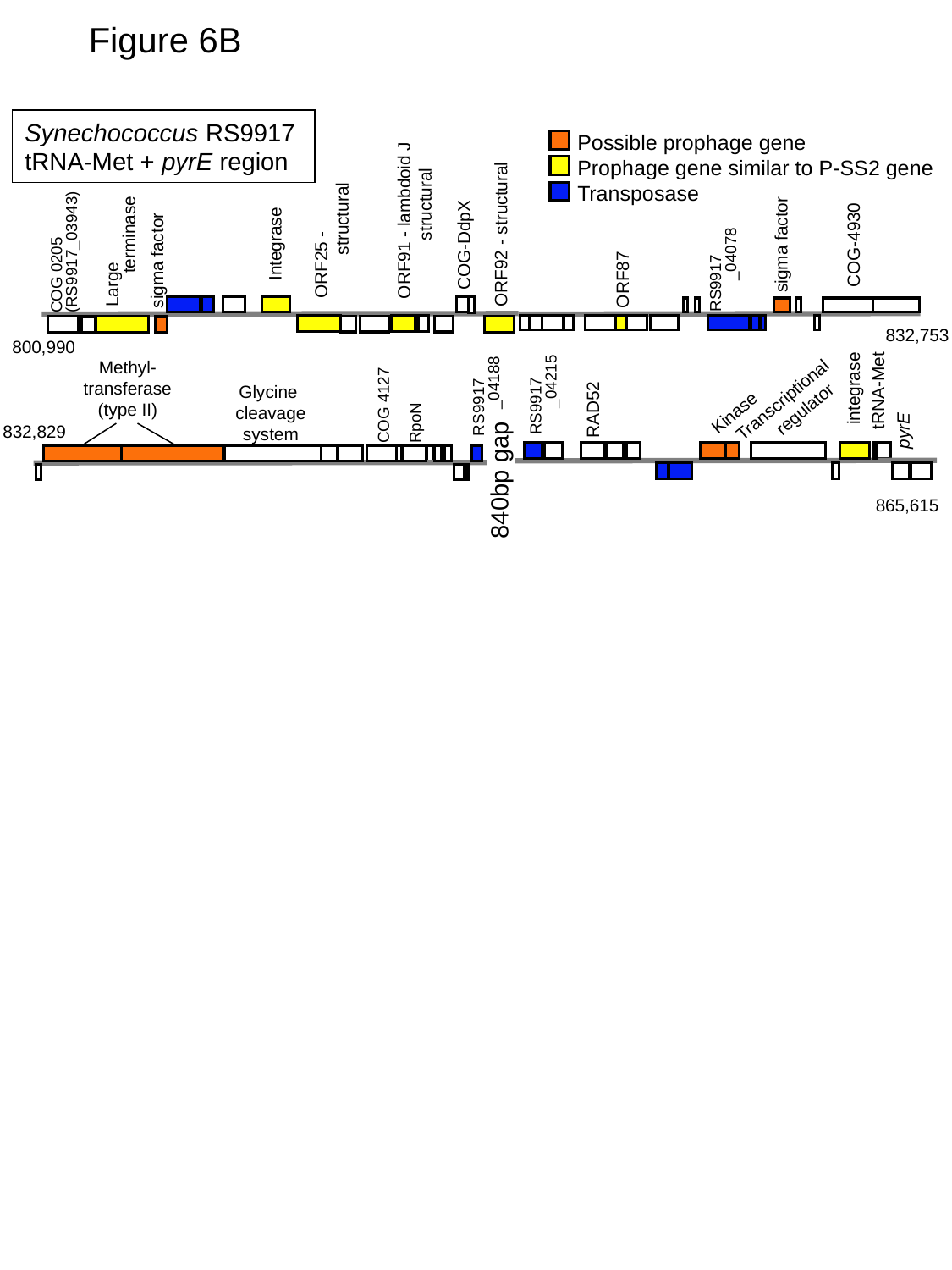

# Figure 6B
Synechococcus RS9917
tRNA-Met + pyrE region
Possible prophage gene
Prophage gene similar to P-SS2 gene
Transposase
ORF91 - lambdoid J
 structural
ORF25 -
 structural
ORF92 - structural
Integrase
sigma factor
Large
 terminase
COG-DdpX
COG-4930
COG 0205
(RS9917_03943)
sigma factor
RS9917
 _04078
ORF87
832,753
800,990
Methyl-
transferase
(type II)
Kinase
 Transcriptional
 regulator
 tRNA-Met
pyrE
integrase
RS9917
 _04188
RS9917
 _04215
COG 4127
RpoN
Glycine
cleavage
system
RAD52
832,829
840bp gap
865,615

## Slide 10
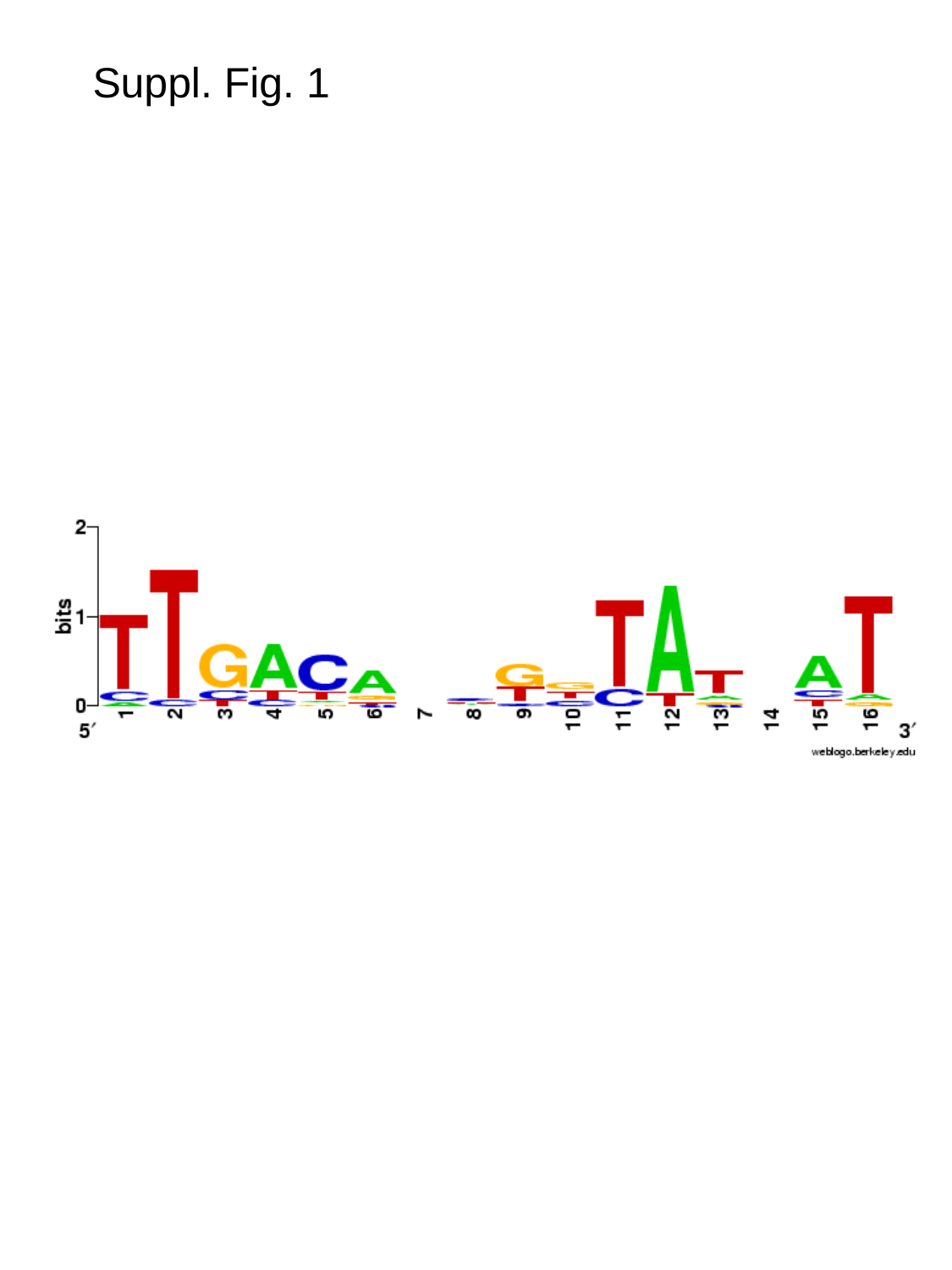

# Suppl. Fig. 1

## Slide 11
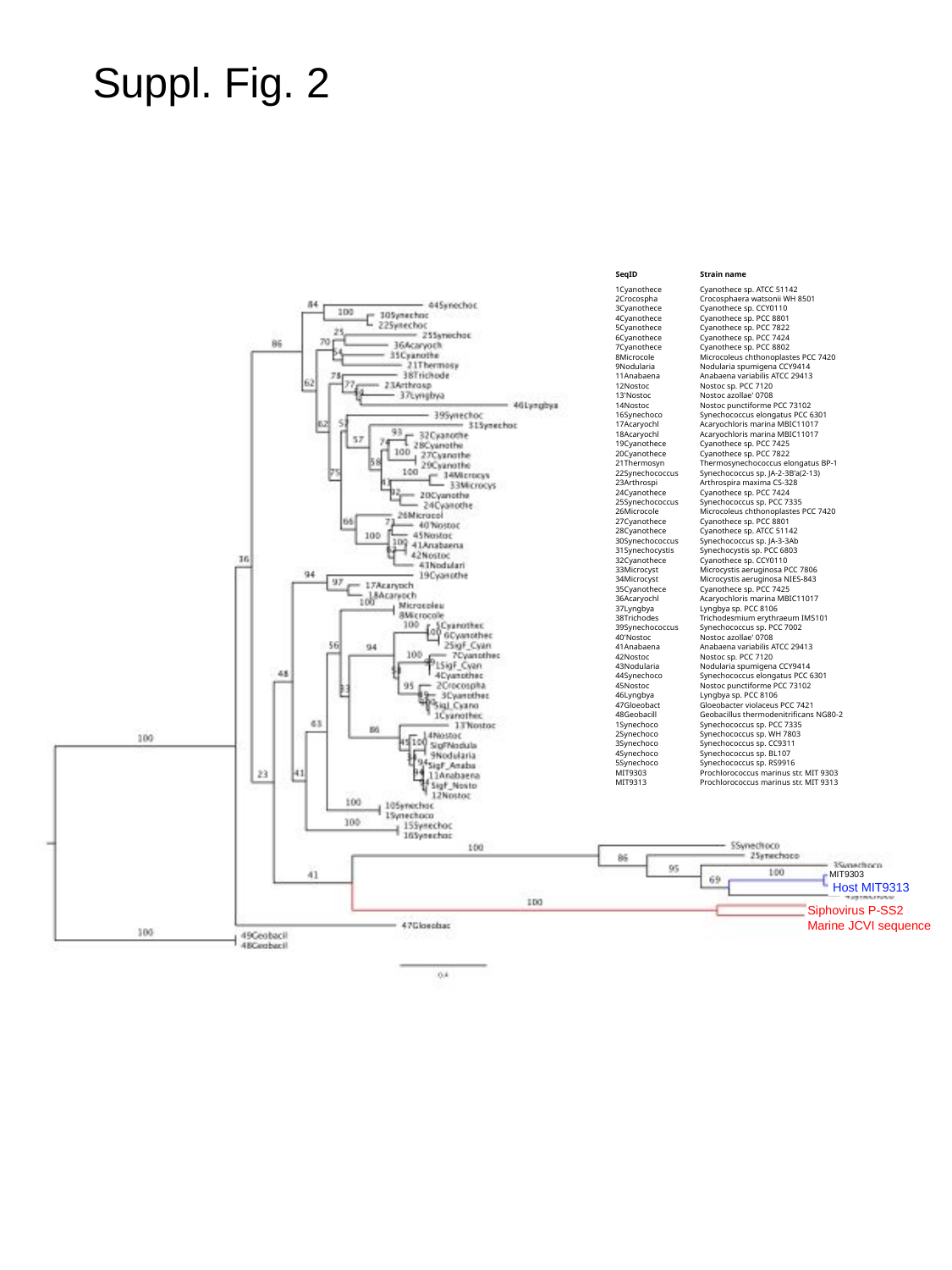

# Suppl. Fig. 2
MIT9303
 Host MIT9313
Siphovirus P-SS2
Marine JCVI sequence

## Slide 12
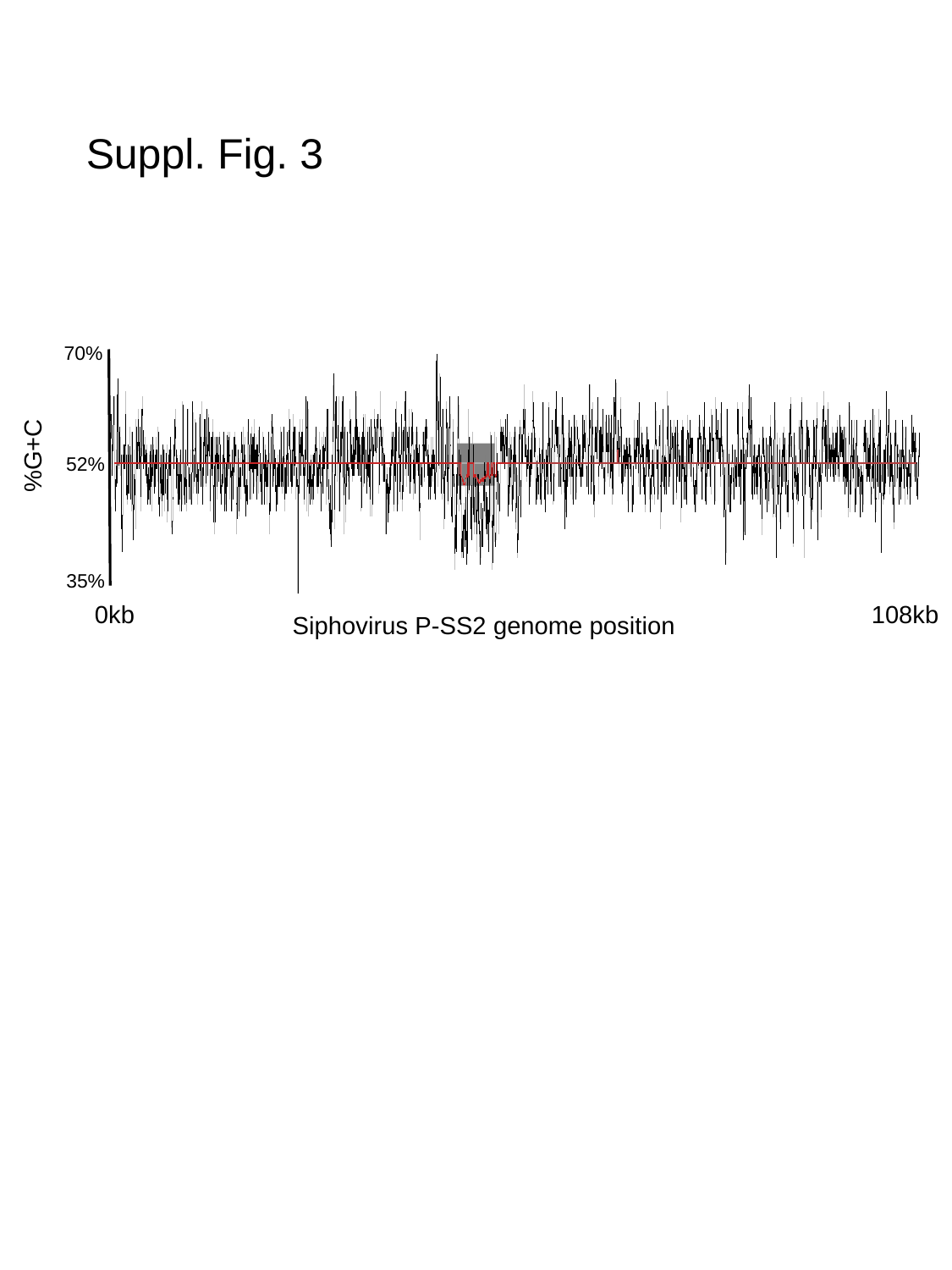

# Suppl. Fig. 3
70%
%G+C
52%
35%
0kb
108kb
Siphovirus P-SS2 genome position
