# Supplementary material for: The genome and structural proteome of an ocean siphovirus: a new window into the cyanobacterial ‘mobilome’
Source: Environ Microbiol. 2009 Nov;11(11):2935–51. doi: 10.1111/j.1462-2920.2009.02081.x (PMC2784084; doi:10.1111/j.1462-2920.2009.02081.x)
Supplement: Supplementary file 2 [file emi0011-2935-SD2.doc]

Supplementary Table 1: Genomic locations of predicted promoters and terminators.

| PROMOTERS | Start | End | strand | -35 seq | -10 seq |
| --- | --- | --- | --- | --- | --- |
| PROM_1 | 2025 | 2053 | + | ttgaca | tggtatcag |
| PROM_2 | 2751 | 2778 | + | ttgaag | ttttttcat |
| PROM_3 | 5658 | 5689 | + | ttctcc | tttttttat |
| PROM_4 | 9431 | 9459 | + | ttgaca | cgccatgat |
| PROM_5 | 10083 | 10111 | + | ttgaca | cgccatgat |
| PROM_6 | 10735 | 10763 | + | ttgaca | cgccatgat |
| PROM_7 | 24917 | 24943 | + | ctgtca | tcttataat |
| PROM_8 | 25784 | 25811 | + | ttgaca | atgtattct |
| PROM_9 | 26384 | 26413 | + | ttgaca | ccctatctt |
| PROM_10 | 26927 | 26955 | + | ttgaca | aggtaactt |
| PROM_11 | 29803 | 29835 | + | ttgata | cggtagaat |
| PROM_12 | 32692 | 32721 | + | tcgtta | gggtagact |
| PROM_13 | 84300 | 84327 | + | tttctt | aggtaattt |
| PROM_14 | 88983 | 89015 | + | ttgctc | aggtagcct |
| PROM_15 | 12296 | 12281 | - | ttgaca | agtcatatt |
| PROM_16 | 12945 | 12930 | - | ttgaca | tgtcatact |
| PROM_17 | 13268 | 13253 | - | ttgaca | ggtcatact |
| PROM_18 | 23168 | 23150 | - | ttaata | tcttatact |
| PROM_19 | 92159 | 92141 | - | ttgcct | cgctatgat |
|  |  |  |  |  |  |
| TERMINATORS | Start | End | strand | sequence |  |
| TERM_1 | 2756 | 2769 | + | gggactacggtccc | |
| TERM_2 | 5662 | 5679 | + | cccccggcccaccgggga | |
| TERM_3 | 6744 | 6759 | + | gcccctcttgaggggc | |
| TERM_4 | 9462 | 9480 | + | gggagggttaagccctccc | |
| TERM_5 | 10114 | 10132 | + | gggagggttaagccctccc | |
| TERM_6 | 10766 | 10784 | + | gggagggttaagccctccc | |
| TERM_7 | 11506 | 11527 | + | gggacgcccctagtcgcgtccc | |
| TERM_8 | 15047 | 15063 | + | gcccctctaggaggggc | |
| TERM_9 | 22802 | 22831 | + | gaccctgggagagtccatgctcctggggtc | |
| TERM_10 | 25749 | 25765 | + | ccccgcctagtgcgggg | |
| TERM_11 | 26113 | 26138 | + | agccctaagggtggtgccttagggcc | |
| TERM_12 | 26893 | 26909 | + | gcccccctcgagggggc | |
| TERM_13 | 27149 | 27162 | + | gggcctacgggccc | |
| TERM_14 | 27886 | 27902 | + | gggctccctaggggccc | |
| TERM_15 | 29772 | 29789 | + | gcctcccgcaaggggggc | |
| TERM_16 | 30163 | 30176 | + | ccccctcgaggggg | |
| TERM_17 | 30228 | 30240 | + | gagagctcctctc | |
| TERM_18 | 53859 | 53874 | + | gggggcttcggccccc | |
| TERM_19 | 84167 | 84186 | + | ggccctgcaacagcagggtc | |
| TERM_20 | 85665 | 85682 | + | gcctcccttacggggggc | |
| TERM_21 | 106155 | 106172 | + | gcccccatatctgggggc | |
| TERM_22 | 107068 | 107084 | + | ggccccttcaaggggcc | |

Supplementary Table 2: Details of genomic regions sharing synteny with representative genomes presented in Fig. 6A.

| ***Isolates*** | ***Genome region (nt position)*** |
| --- | --- |
| *Same genome arrangement as MED4* | |
| MIT9215 | 253,135 - 275,635 |
| AS9601 | 256,220 - 278,720 |
| MIT9211 | 267,649 - 290,149 |
| MIT9301 | 255,803 - 278,720 |
| MIT9312 | 246,647 - 269,147 |
| MIT9515 | 265,543 - 288,043 |
| NATL1A | 309,245 - 331,745 |
| NATL2A | 291,439 - 313,939 |
| SS120 | 280,207 - 302,707 |
| *Same genome arrangement as MIT9313* | |
| MIT9303 | 2,174,404 - 2,196,904 |
| *Same genome arrangement as WH8102* | |
| BL107 | 72,445 - 94,945 |
| CC307 | 1,997,682 - 2,020,182 |
| CC9311 | 283,817 - 306,317 |
| CC9605 | 235,407 - 257,907 |
| CC9902 | 277,436 - 298,686 |
| RS9916 | 57,065 - 79,565 |
| WH7803 | 302,592 - 324,992 |
| WH7805 | 220,710 - 252,960 |

Supplementary Table 3: Environmental sequence reads from the Global Ocean Survey (Rusch et al. 2007) that were best hits to the P-SS2 genome.

| **Read name** | **GOS site** | **e-value** | **Alignment length (bp)** |
| --- | --- | --- | --- |
| JCVI_READ_1091145058945 | GS004 | 2.03E-08 | 47 |
| JCVI_READ_1091145481933 | GS004 | 2.21E-08 | 47 |
| JCVI_READ_1091141253121 | GS015 | 1.22E-31 | 242 |
| JCVI_READ_1095522140668 | GS033 | 3.04E-07 | 73 |
| JCVI_READ_1092961193753 | GS051 | 1.23E-06 | 72 |
| JCVI_READ_1108830208947 | GS114 | 4.41E-09 | 40 |
| JCVI_READ_1105297207090 | GS110b | 5.58E-06 | 95 |
